# Leveraging 3d‐4f Coordination: Molecular Quantum Spring‐Magnet Behavior in Axial Ni_2_Ln Complexes

**DOI:** 10.1002/anie.202522076

**Published:** 2025-12-19

**Authors:** Zhaoyang Jing, Eufemio Moreno‐Pineda, Sagar Paul, Appu Sunil, Olaf Fuhr, Yaorong Chen, Wolfgang Wernsdorfer, Mario Ruben

**Affiliations:** ^1^ Institute of Nanotechnology (INT) Karlsruhe Institute of Technology (KIT), Hermann‐von‐Helmholtz‐Platz 1 D‐76344 Eggenstein‐Leopoldshafen Germany; ^2^ Physikalisches Institut (PHI) Karlsruhe Institute of Technology (KIT) Physikhochhaus, Geb. 30.23, Wolfgang‐Gaede‐Str. 1 D‐76131 Karlsruhe Germany; ^3^ Depto. de Química‐Física Universidad de Panamá, Facultad de Ciencias Naturales, Exactas y Tecnología Panamá 0824 Panamá; ^4^ Grupo de Materiales Universidad de Panamá, Facultad de Ciencias Naturales, Exactas y Tecnología Panamá 0824 Panamá; ^5^ Karlsruhe Nano Micro Facility (KNMFi) Karlsruhe Institute of Technology (KIT) Kaiserstraße 12 Karlsruhe 76131 Germany; ^6^ Institute of Quantum Materials and Technologies (IQMT) Karlsruhe Institute of Technology (KIT) Hermann‐von‐Helmholtz‐Platz 1 D‐76344 Eggenstein‐Leopoldshafen Germany; ^7^ Centre Européen de Sciences Quantiques (CESQ) Institut de Science et d'Ingénierie Supramoléculaires (ISIS) 8 allée Gaspard Monge, BP 70028 Strasbourg Cedex 67083 France

**Keywords:** 3d–4f Exchange coupling, Exchange‐spring behavior, Magnetic bistability, Quantum tunneling of magnetization (QTM), Single‐molecule magnets

## Abstract

We report heterotrimetallic 3d–4f complexes, mimicking classical exchange spring magnets at the molecular scale. The complexes feature a linear Ni···Ln···Ni core, where the lanthanide ion is sandwiched between two Ni^2+^ centers coordinated by N_3_O_3_ ligand environments. The complexes are isostructural, while CASSCF calculations reveal collinear anisotropy axes and favorable electronic configurations for magnetic bistability in selected systems. Magnetic characterization via DC, AC, and µSQUID magnetometry down to 30 mK demonstrates slow magnetic relaxation and open hysteresis loops exclusively in **Ni_2_Tb**, **Ni_2_Dy**, and **Ni_2_Ho**. These systems exhibit ferromagnetic 3d‐4f coupling, while their isolated or antiferromagnetically coupled analogs (**Ni_2_Y**, **Zn_2_Tb**/**Dy**) and **Ni_2_Er**/**Yb** counterparts show fast relaxation and closed loops. Analysis suggests that the Ni^2+^ ions alone, with modest anisotropy, deviate from the expected “hard” magnetic behavior due to a broad zero‐field QTM, while the Ln^3+^ ions alone serve as the “soft” phase with large magnetic moments and sharp zero‐field QTM. Nevertheless, when brought together, their coupling and alignment of the anisotropy axis enhances the magnetic performance with exchange‐bias features mimicking the macroscopic exchange spring magnets. We highlight an optimal utilization of 3d‐4f coordination in designing molecular magnets with tunable relaxation and bistability, advancing prospects for quantum information and nanoscale magnetic devices.

## Graphical abstract

We report heterotrimetallic **Ni_2_Ln** complexes mimicking exchange spring magnets at the molecular level. CASSCF calculations show collinear anisotropy axes, while µSQUID studies reveal slow relaxation and open hysteresis in **Ni_2_Tb**, **Ni_2_Dy**, and **Ni_2_Ho**. Ferromagnetic 3d–4f coupling and anisotropy alignment enhance bistability, offering promise for quantum, and nanoscale magnetic devices.

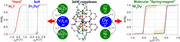

## Introduction

**Scheme 1:**
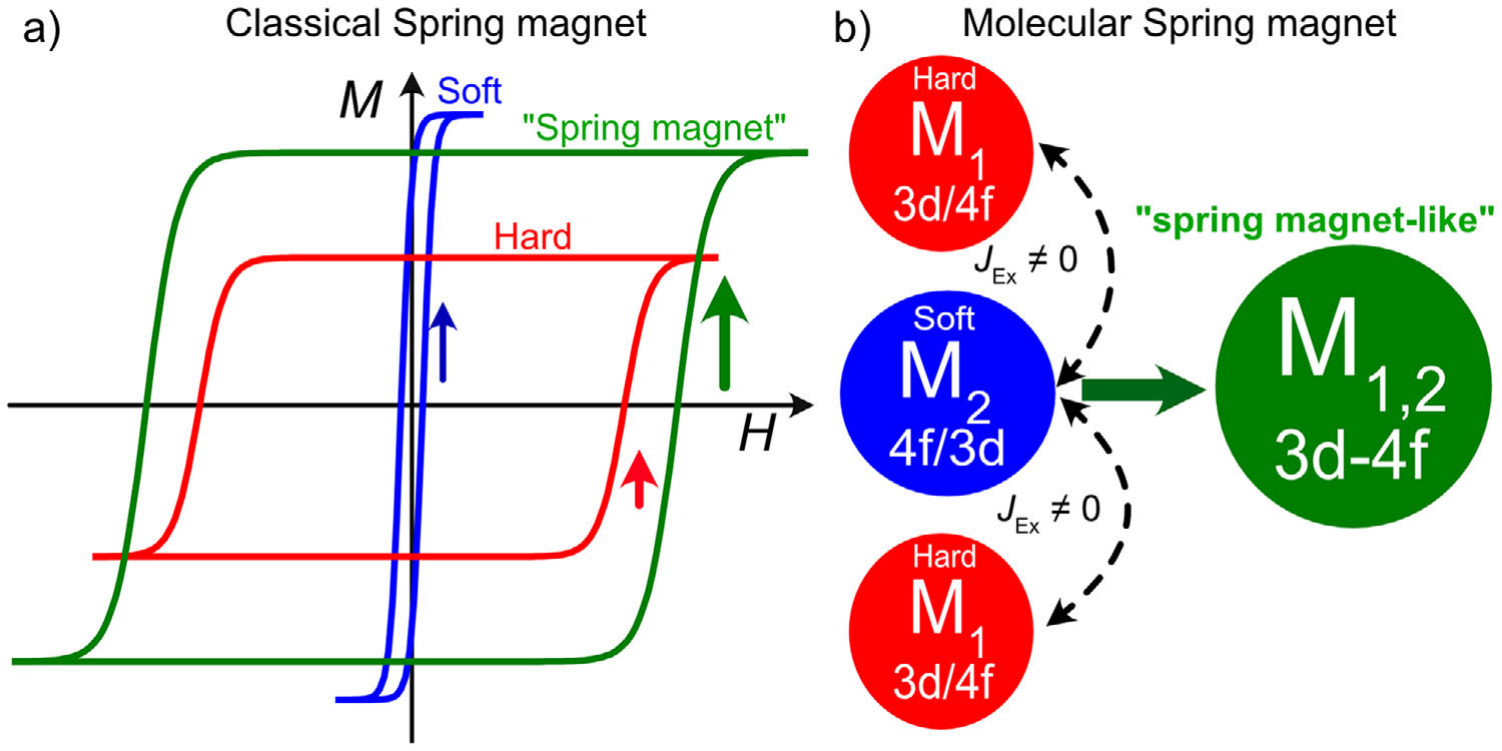
a) Schematic representation of a classical spring magnet. The “soft” phase imparts the intensity to the overall *M*(*H*) signal, while the “hard” phase offers a large anisotropy with a wide opening. The results “spring magnet” behavior results from the exchange couple character of the “soft” and “hard” phase, leading to an open loop with enhanced intensity. b) Molecular “Spring magnet” analog, in which 3d and 4f ions are brought together and, through an exchange behavior, result in an overall improved performance compared to the isolated 3d/4f analog.

A classical exchange spring magnet is a composite magnetic material that integrates a hard magnetic phase with a soft magnetic phase to optimize performance.^[^ ^1^ ^]^ The hard phase imparts high coercivity, enabling resistance to demagnetization, while the soft phase contributes to achieving a large magnitude of saturation magnetization, thereby enhancing the overall magnetic strength (Scheme 1a). These phases are tightly coupled via long‐range interactions, allowing the soft phase to “spring back” into alignment with the “hard phase” when subjected to an external magnetic field. This synergistic behavior yields magnets with superior energy products, making them highly efficient for applications in electric motors, magnetic recording, and spintronic devices.^[^ ^1^, ^2^, ^3^ ^]^ Examples of exchange‐spring magnets are Nd_2_Fe_14_B/α‐Fe, SmCo_5_/Fe, and BaFe_12_O_19_/Fe_3_O_4_ composites, which combine a hard magnetic phase for coercivity with a soft magnetic phase for high magnetization through interfacial exchange coupling.^[^ ^1^, ^2^, ^3^ ^]^ The molecular quantum analog of spring‐magnets is likewise desired; however, the lack of synthetic strategies to couple two counter parts while avoiding large quantum tunneling rates under transverse magnetic interactions and direct relaxation rates usually prohibits its observation.

In this context, a molecular spring magnet should also consist of a “soft” and “hard phase”, with the right interaction yielding an overall unison magnetic behavior. To achieve this goal, two strategies might be envisioned. The first one consists of creating heterometallic 4f complexes, where one of the ions provides the hardness to the system, while the other one delivers the softness. Unfortunately, given that the interaction between 4f ions is often of dipolar origin (hence limited interaction strength), accounting for the buried 4f shell, this approach is less favorable. The second approach consists of leveraging the often‐observed ferromagnetic interactions in 3d‐4f complexes.^[^ ^4^ ^]^ In contrast to the inner nature of 4f elements, 3d orbitals are more delocalized, allowing the electrons to engage in (stronger) exchange interaction, as it would be desirable (Scheme 1b). However, challenges remain to reduce transverse interactions in such an approach. Stronger axial interionic interactions are often accompanied by enhanced transverse components, which compromise the system's quantum memory by enlarging zero‐field quantum tunneling gaps.

Early multinuclear 3d‐based single‐molecule magnets (SMMs) demonstrated magnetic bistability, driven by strong metal–metal interactions and collinear anisotropy axes.^[^ ^5^, ^6^ ^]^ These features led to giant spin ground states, which effectively suppressed zero‐field quantum tunneling of magnetization (QTM) by minimizing multibody tunneling events, resulting in open magnetic hysteresis loops.^[^ ^7^, ^8^ ^]^ Furthermore, this approach seems appropriate also for multinuclear 4f systems, given that even the best‐performing single lanthanide 4f‐SMMs continue to suffer from pronounced zero‐field QTM, posing a challenge for their practical implementation in data storage technologies.^[^ ^9^, ^10^, ^11^, ^12^ ^]^ Recent work by some of us has demonstrated that one of the most effective strategies to mitigate the notorious zero‐field (ZF) QTM is the coupling of polymetallic lanthanide systems, which can yield exceptionally long QTM relaxation times. Notable examples include di‐,^[^ ^13^, ^14^, ^15^ ^]^ tri‐,^[^ ^16^ ^]^ tetra‐,^[^ ^17^, ^18^, ^19^ ^]^ hexa,‐^[^ ^20^, ^21^, ^22^, ^23^, ^24^ ^]^ and octa‐nuclear complexes.^[^ ^25^ ^]^

Herein, we explore a 3d/4f approach, where, through a combination of 3d and 4f metals within a single molecule, we exploit the delocalized nature of 3d electrons to promote stronger exchange interactions, while harnessing a large magnitude of magnetization from the lanthanide ions. Our strategy leverages the system's axial architecture to suppress transverse components in the spin Hamiltonian, thereby optimizing the magnetic profile arising from 3d/4f coordination. In this work, we investigate a series of M^2+^–Ln^3+^ complexes (M = Ni^2+^, Zn^2+^; Ln = Dy^3+^, Tb^3+^, Ho^3+^) using µSQUID magnetometry down to sub‐kelvin temperatures. These complexes feature linear heterotrimetallic architectures, with the lanthanide ion sandwiched between two magnetic transition metals M^2+^ centers. When one of the paramagnetic ions is replaced by its diamagnetic analog Y^3+^ or by Zn^2+^, no or a diminished magnetic bistability is observed. In contrast, when both paramagnetic ions are present, well‐defined open hysteresis loops emerge. Analogous to exchange spring magnets, the high anisotropy of one magnetic center is coupled to a softer magnetic phase with a larger magnetic moment, resulting in enhanced magnetic performance. Our findings reveal that by leveraging 3d–4f interactions, it is possible to construct molecular systems with improved magnetic characteristics, even when the individual 3d or 4f precursors exhibit fast relaxation dynamics.

## Results and Discussion

### Structure and Electronic Properties

**Figure 1:**
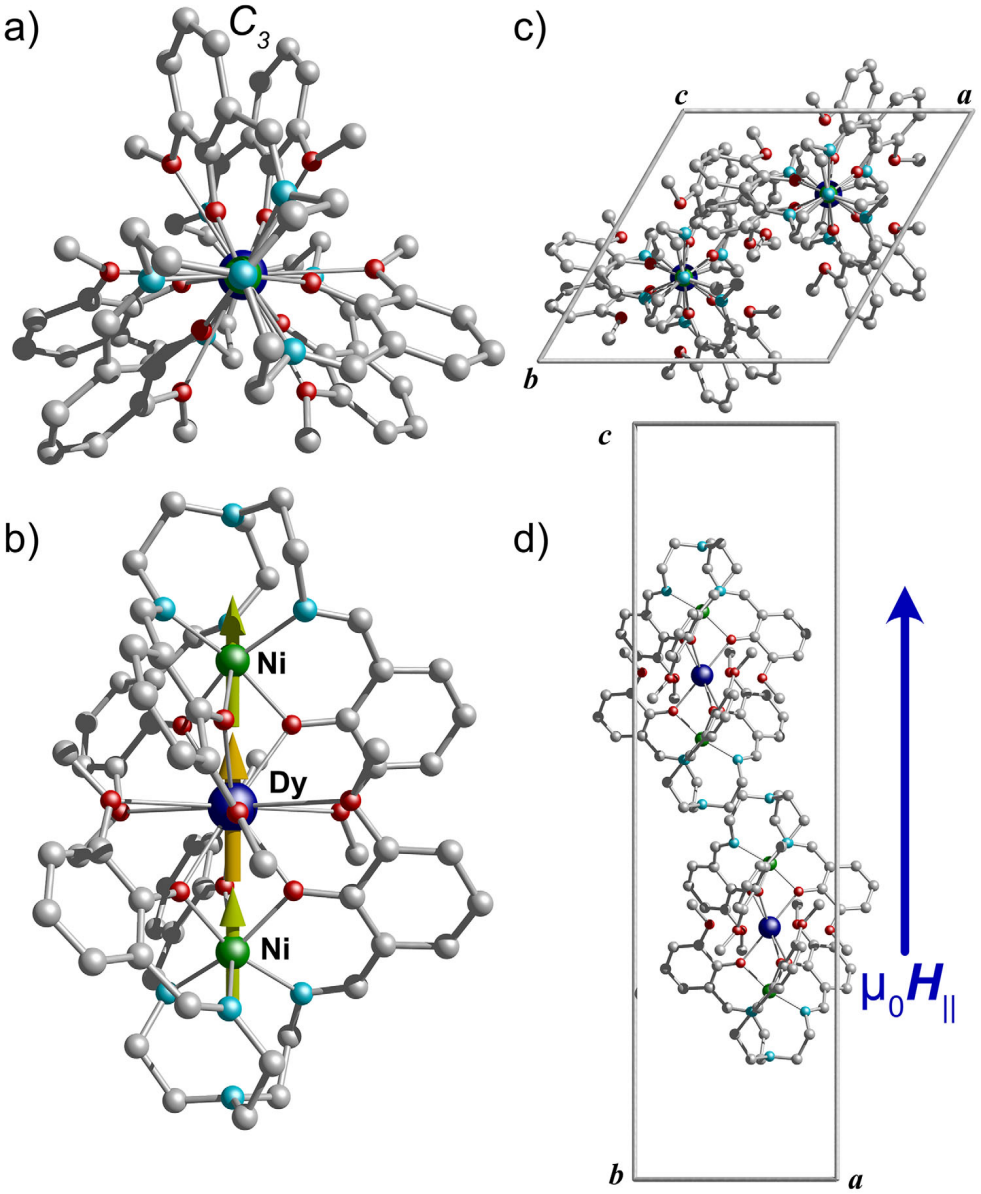
a) Crystal structure of **Ni_2_Dy** viewed along the *C_3_* axis and b) perpendicular to it. The Yellow and green arrows are the anisotropy axes for the Dy^3+^ and Ni^2+^ ions in the complex, as obtained from CASSCF calculations. Unit cell highlighting the colinear arrangement of the **Ni_2_Dy** molecules within the unit cell, viewed along the c) *c*‐axis and d) *b*‐axis. The blue arrow represents the magnetic field direction along the easy axis of the crystal during the µSQUID investigations. Color code: Dy, blues; Ni, green; N, cyan; O, red; C, grey. Hydrogens are omitted for clarity.

The synergistic effect of 3d‐4f interactions was investigated in a series of linear **Ni_2_Ln** complexes, where Ln = Tb^3+^, Dy^3+^, Ho^3+^, Er^3+^, and Yb^3+^ (Figure 1). All complexes are isostructural and crystallize in the trigonal *R*32 space group,^[^ ^26^ ^]^ with three molecules per unit cell, each exhibiting a colinear arrangement (Figure 1c,d).^[^ ^26^, ^27^, ^28^ ^]^ The following structural description focuses on the **Ni_2_Dy** complex.

In **Ni_2_Ln**, two deprotonated H_3_L ligands [H_3_L = Tris(((2‐hydroxy‐3‐methoxybenzyl)‐amino)ethyl)amine] encapsulate one Ni^2+^ ion within an inner N_3_O_3_ coordination pocket, formed by three nitrogen atoms from imine groups and three oxygen atoms from phenoxide moieties. The resulting [NiL]^−^ units flank a central Dy^3+^ ion, yielding a linear Ni···Dy···Ni metallic core with the formula [Ni_2_DyL_2_]^+^ (Figure 1a,b). Six uncoordinated methoxy groups in the equatorial plane surround the Dy^3+^ ion, effectively shielding it from further coordination. Bond distances within the complex include Ni–N: 2.090(4) Å, Ni–O: 2.062(3) Å, and Dy–O: 2.346(3) Å, with the shortest intramolecular Dy···Ni separation measured at 3.2442(8) Å. Considering only the metal centers, a *C*_3_ rotation axis passes through the Ni–Dy–Ni core, accompanied by three perpendicular *C*_2_ axes.

Each Ni^2+^ ion adopts a distorted octahedral geometry, defined by two staggered equilateral triangular faces—one composed of three nitrogen atoms (N_3_), the other of three oxygen atoms (O_3_) (Table S6). The coordination environment around Dy^3+^, formed by six oxygen atoms (O_6_), deviates significantly from a regular octahedron, as indicated by continuous shape measures (CShM),^[^ ^29^ ^]^ and is better described as an axially elongated, distorted trigonal antiprism (Table S7).

Intermolecular distances between neighboring molecules—Ni···Ni: 9.321 Å, Dy···Ni: 11.874 Å, and Dy···Dy: 11.764 Å—are sufficiently long to suggest negligible intermolecular Dy···Dy and Dy···Ni interactions compared to intramolecular ones. A triflate counterion in the lattice charge balances the complexes. Notably, no hydrogen bonding or other weak interactions are observed between the trinuclear cations and the counterions in any of the complexes.

A key feature of this system, crucial for subsequent magnetic investigations, is its modularity: either of the 3d or 4f magnetic ions can be selectively replaced with diamagnetic ions—Zn^2+^ or Y^3+^, respectively, yielding isostructural “soft‐like” or “hard‐like” counterparts. The lanthanide ions can be readily exchanged, including substitution with diamagnetic Y^3+^ to form the **Ni_2_Y** complex. Conversely, Ni^2+^ can be replaced with diamagnetic Zn^2+^, affording **Zn_2_Ln** analogs (Ln = Dy^3+^, Tb^3+^), while preserving the overall structural framework and unit cell parameters. This modularity enables a systematic comparison of the electronic and magnetic properties across the **Ni_2_Y**, **Ni_2_Ln**, and **Zn_2_Ln** series, highlighting the enhanced magnetic features intrinsic to the **Ni_2_Ln** complexes. Note that such molecular motifs have also been reported for several other paramagnetic 3d and 4f ions^[^ ^27^, ^28^, ^30^, ^31^, ^32^, ^33^, ^34^, ^35^, ^36^, ^37^ ^]^; however, herein we focus on a systematic comparison of the 3d/4f interaction effect, taking advantage of the modularity of the complex, in the temperature regime where the 3d/4f interaction effect is discernible.

The electronic structure of the individual ions in the **Ni_2_Ln** complexes (and the counterparts: **Ni_2_Y** and **Zn_2_Ln**) was investigated using the Complete Active Space Self‐Consistent Field (CASSCF) method.^[^ ^38^, ^39^, ^40^ ^]^ The calculations for the **Ni_2_Ln** series predict a collinear, head‐to‐tail arrangement of the magnetic easy axes for both Ni^2+^ and Ln^3+^ centers (see green and yellow arrows in Figure 1b), thereby maximizing ferromagnetic dipolar interactions. This alignment is particularly significant given the localized nature of 4f orbitals, which predominantly engage in dipolar rather than exchange interactions.^[^ ^41^, ^42^ ^]^

For Ni^2+^, the axial ligand field parameter ($B_{2}^{0}$) range from −0.5 to −1.1 cm^−1^ across the series, with negligible rhombic ($B_{2}^{2}$) components (∼0 cm^−1^). A gradual increase in $\;B_{2}^{0}$ of Ni^2+^ for Tb^3+^ to Yb^3+^ is observed, consistent with the lanthanide contraction, which imposes greater axiality on the Ni^2+^ coordination environment (Table S10 and Figure S4). A relatively large and negative $B_{2}^{0}$ characterizes the Tb^3+^, Dy^3+^, and Ho^3+^ systems, consistent with an axial anisotropy favorable for oblate ions (Table S18 and Figure S4 (2^nd^ axis)). In the case of Dy^3+^, the predicted electronic structure in both **Ni_2_Dy** and **Zn_2_Dy** reveals mixed wavefunctions (∼90% |±15/2⟩) and a small energy gap between the ground and first excited states (51 cm^−1^ for **Ni_2_Dy** and 40 cm^−1^ for **Zn_2_Dy**), suggesting limited anisotropy (Tables S12, S17). In contrast, Tb^3+^ exhibits a pure ground state composition (>99% |±6⟩) and a significantly larger energy separation (>200 cm^−1^), indicative of strong axial anisotropy (Tables S11, S17). Noteworthy, the highly axial character of the Ni^2+^ ion (*E* = 0) allows us to investigate the 3d/4f interaction effect, with reduced transverse field effects, in turn promoting slower tunneling rates^[^ ^30^, ^31^, ^32^ ^]^

For Ho^3+^, the wavefunction is composed mainly of |±7⟩ (82%) and |±4⟩ (18%) states with a separation of ∼74 cm^−1^ between the ground and first excited state (Table S13). However, the tunneling gaps in the ground state are present in both Tb^3+^ and Ho^3+^ systems (0.018–0.062 cm^−1^), potentially contributing to fast relaxation dynamics. For Er^3+^ and Yb^3+^, the $B_{2}^{0}$ parameters are positive, signifying that the largest anisotropic component is of easy‐plane type, as expected for prolate ions. Furthermore, we find the ground‐state wavefunctions are more mixed, indicating reduced magnetic anisotropy and a lower likelihood of slow relaxation behavior (Tables S14, S15). Despite the large negative $B_{2}^{0}$ in Tb^3+^, Dy^3+^, and Ho^3+^, the presence of the transverse ligand field parameter (Table S18) and wave function mixing causes the **Zn_2_Ln** systems to feature the “soft‐like” phase, i.e., a large magnetization with a sharp zero‐field QTM step in *M*(*H*) curves (see later), as usually observed in lanthanide monomer systems.

**Figure 2:**
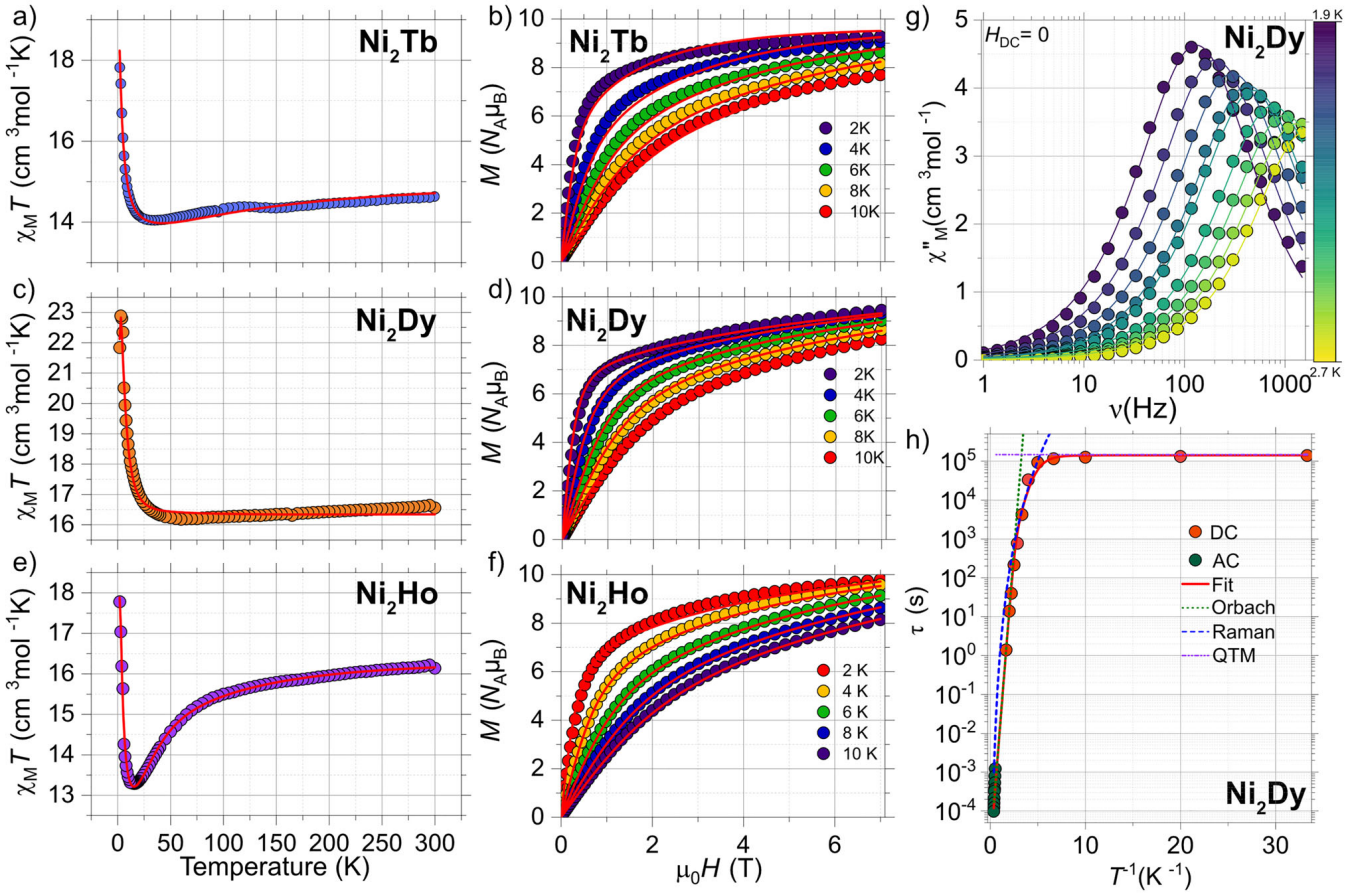
Temperature‐dependent molar magnetic susceptibility study (χ_M_ *T*(*T*)) for a) **Ni_2_Tb**, c) **Ni_2_Ho**, and e) **Ni_2_Dy**. The experimental data (open circles) highlight the existence of ferromagnetic interactions operating within the complexes. Panels b), d), and f) show the magnetization data from 0 to 7 T and for temperatures in the range between 2 and 10 K, for **Ni_2_Tb**, **Ni_2_Dy**, and **Ni_2_Ho**, respectively. The solid lines in these figures are the best fits utilizing the Equation (2) and the parameters as described in the text. Panel g) shows the frequency‐dependent magnetic susceptibility. The solid lines are the best fit to a generalized Debye model. h) Shows the temperature dependence of the relaxation time (τ(*T*)) obtained from AC and DC (µSQUID) data. The solid lines are the best fit to Equation (1).

Static magnetic measurements of the **Ni_2_Ln** series on bulk powder samples confirm the presence of two Ni^2+^ ions and one Ln^3+^ center per molecule, as inferred from the room‐temperature χ_M_ *T* values (Table S8). Upon cooling, the χ_M_ *T*(*T*) products for **Ni_2_Tb**, **Ni_2_Dy**, and **Ni_2_Ho** exhibit a pronounced upturn, consistent with ferromagnetic coupling between the metal centers (Figure 2a–c).^[^ ^28^, ^30^, ^31^, ^32^, ^33^, ^34^, ^35^, ^36^, ^37^, ^43^ ^]^ In contrast, the χ_M_ *T*(*T*) values for **Ni_2_Er** and **Ni_2_Yb** decrease with temperature, indicative of weak or negligible ferromagnetic interactions and the thermal depopulation of Stark sublevels (Figure S5). Field‐dependent magnetization measurements, *M*(*H*), for **Ni_2_Tb**, **Ni_2_Dy**, and **Ni_2_Ho** yield *M*(*H*) values at the lowest temperature (2 K) and highest field (7 T) of 9.3, 9.4, and 8.9 *N*_A_µ_B_, respectively. For the **Ni_2_Er** and **Ni_2_Yb** complexes, we find the largest *M*(*H*) values at 2 K and 7 T of 9.8 and 5.7 *N*_A_µ_B_, respectively.

To probe the anisotropic character and dynamic magnetic behavior, alternating current (AC) susceptibility measurements were performed. Frequency‐ and temperature‐dependent studies reveal an out‐of‐phase component (χ″_M_) only for **Ni_2_Dy** at zero field, indicating an SMM behavior (Figure 2d). Simultaneous fitting of both the in‐phase (χ′_M_) and χ″_M_ components to a generalized Debye model enables extraction of the temperature‐dependent relaxation times τ(*T*) (green symbols in Figure 2h). No SMM features down to 2 K were observed for the other **Ni_2_Ln** complexes or for any of the **Zn_2_Ln** analogs.

### µSQUID Investigation at Sub‐Kelvin Temperatures

**Figure 3:**
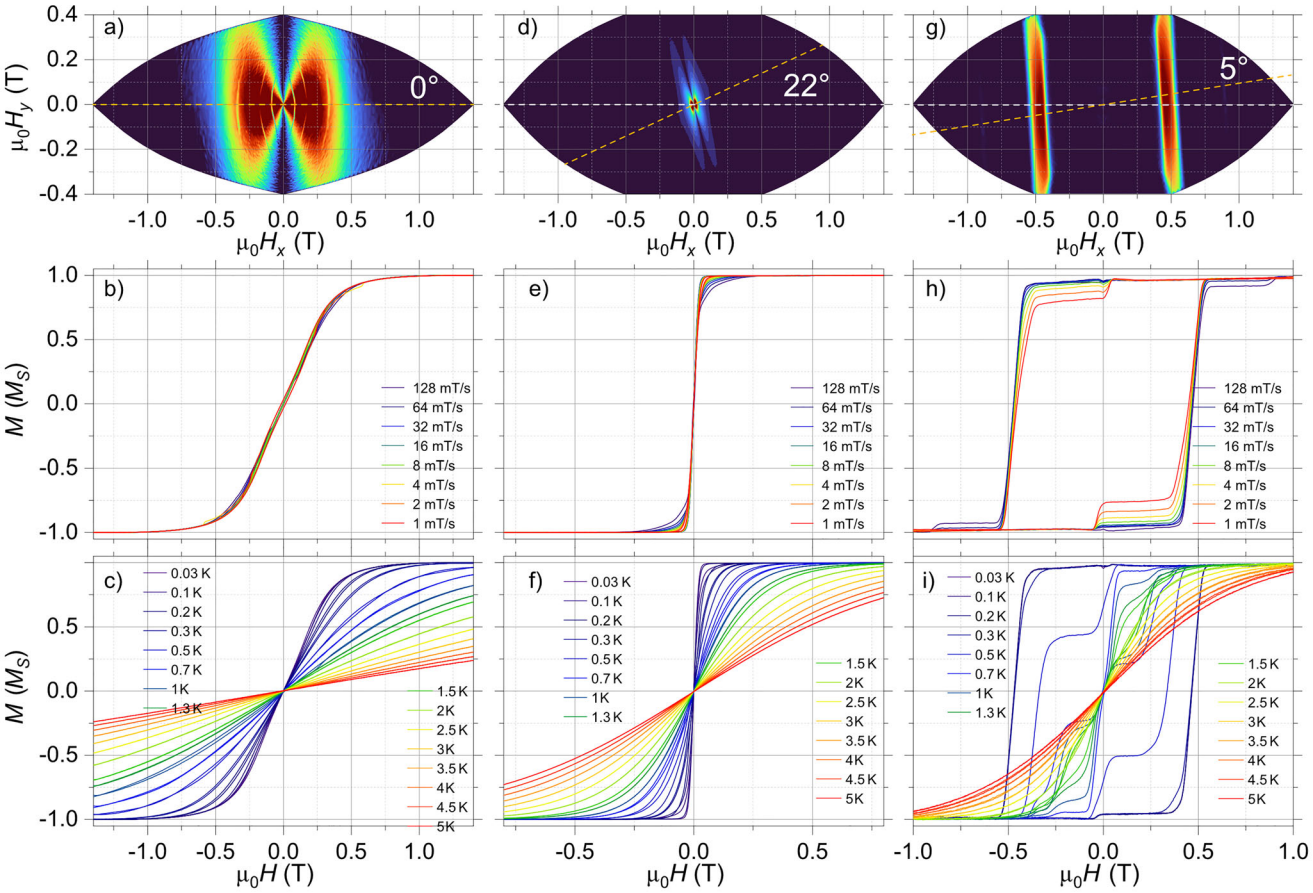
Angle dependence of the M(H) loops, by mapping the corresponding derivatives δ*M*/δ*H*, obtained employing µSQUID arrays for single crystals of a) **Ni_2_Y**, d) **Zn_2_Dy**, and g) **Ni_2_Dy**. The data were collected at a fixed sweep rate of δ*H*/δ*t = *64 mT/s and at a base temperature of 30 mK. The dashed orange line in these maps highlights the easy axis of the crystal with respect to the µ_0_ *H_x_* magnet. b), e), and h) Panels show the sweep‐rate dependent *M*(*H*) loops at a base temperature of 30 mK for **Ni_2_Y**, **Zn_2_Dy**, and **Ni_2_Dy**, respectively. c), f), and i) Panels contain the temperature‐dependent hysteresis loops with a fixed sweep rate δ*H*/δ*t = *16 mT/s. The data collected in panels (d) i) were collected with the magnetic field aligned along the easy axis of the crystal, as determined in the angular maps shown in (a), (d), and (g).

Magnetization measurements conducted at even lower temperatures unveil remarkable insights concerning drastically enhanced magnetic properties in some of the **Ni_2_Ln** compounds, compared to their “3d‐ or 4f‐only” counterparts. We discuss these observations in terms of the **Ni_2_Dy** system and its counterparts and later compare it with the analogs. For these sub‐Kelvin studies, we employed the highly sensitive µSQUID magnetometry technique, equipped with a 3D vector magnet with an angular resolution of 0.1°, integrated into a dilution refrigerator capable of temperatures down to 30 mK.^[^ ^44^ ^]^ Single micro‐crystals (10–50 µm) were positioned near the µSQUID loops, and the easy‐axis of the compounds was obtained using the transverse field method and the derivative angular maps (Figure 3a,d,g).^[^ ^45^ ^]^

First, the low‐temperature magnetic measurements in **Ni_2_Y**, along the identified easy axis (see the derivative‐angle map in Figure 3a), revealed narrow hysteresis loops with a small inflection around ±0.15 T, indicative of antiferromagnetic interactions (Figure 3b,c, and Table S20). The crossing at ±0.15 T allows estimation of the mean exchange field (*H*_ex_ = −2*J*_ex_ *m_J_*/*g*µ_B_), yielding 2*J*_ex_ = −0.14 cm^−1^, consistent with values obtained from DC SQUID magnetometry (Table S20 and Figure S5). The observed small hysteresis (at large sweep rates in Figure 3b) suggests that its supposedly “hard‐like” phase is strongly quenched by transverse interactions promoting zero‐field QTM or other (direct) relaxation pathways, eventually leading to a broad transition between ±1 T. Overall, this system does not exhibit remarkable magnetic properties. In fact, at the single‐molecule level and within quantum regimes, referring to it as a “hard” magnet would be an overstatement.

Next, the low‐temperature and sweep‐rate‐dependent studies in **Zn_2_Dy** (along with the identified easy axis, see Figure 3d) showed closed loops and very fast relaxation at zero field (Figure 3e,f), confirming the absence of SMM behavior. The sharp zero‐field transition in *M*(*H*) indicates an efficient Landau‐Zener transition (QTM), and together with the large magnetization magnitude (strong signal detected by µSQUIDs), it qualifies such a system to be a molecular “soft” magnet.

Despite quenched hysteresis in both these counterparts, as the 3d and 4f ions are placed in proximity, as in the **Ni_2_Dy** complex, a large hysteresis (with a strong signal detected by µSQUIDs) appears in the low temperature *M*(*H*) along the identified easy axis (see Figure 3g) as well as for a range of applied field‐directions. The sweep‐rate dependent *M*(*H*) curves at 30 mK (see Figure 3h) reveal a few resonant QTM positions, such as the jumps are observed at 0, ±0.5, and ±0.88 T, and to our general interest, indicate very slow relaxation. Moreover, upon increasing temperature, the *M*(*H*) loops remained hysteretic up to 2 K (Figure 3h), indicating the presence of a barrier to magnetization relaxation and confirming SMM behavior. The observed large open loops allow the determination of their τ(*T*) data at zero‐field (upon returning from saturation fields), for temperatures between 30 and 600 mK (orange symbols in Figure 2h). The τ(*T*) data can be fitted to Equation (1):

(1) $$\tau^{-1}=\tau_{0}^{-1}\;\exp\left(-\frac{U_{\mathrm{eff}}}{k_{B}T}\right)+CT^{n}+\tau_{\mathrm{QTM}}^{-1}$$

comprising the Orbach, Raman, and QTM relaxation, respectively. The best fits yield *U*_eff_ = 10(3) K, τ_0_ = 9.9(4)×10^−6^ s, *C* = 2(2) s, *n* = 7.4(5), and τ_QTM_ = 1.5(5)×10^5^ s. Notably, the QTM time is comparable to the largest reported values for 3d‐4f coordination molecules as well as any SMM (Table S21).^[^ ^11^, ^13^, ^14^, ^15^, ^20^, ^46^, ^47^ ^]^

**Figure 4:**
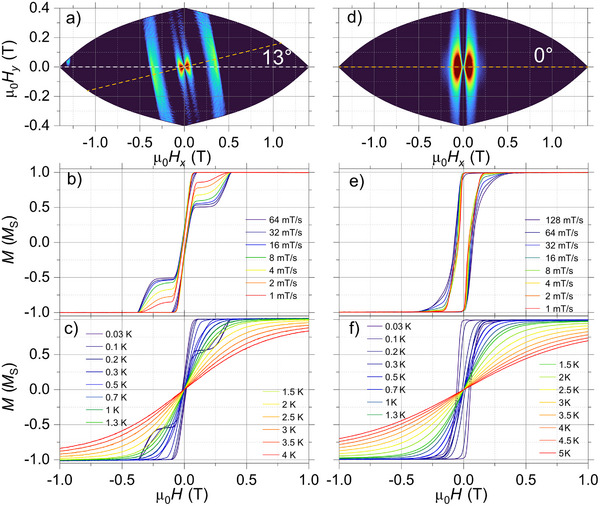
Angle dependence of the *M*(*H*) loops obtained employing µSQUID arrays with varying angles with respect to the crystals for a) **Ni_2_Tb** and d) **Ni_2_Ho**. The data were collected with δ*H*/δ*t = *64 mT/s and at a base temperature of 30 mK. The dashed orange line in these maps highlights the easy axis of the crystal with respect to the µ_0_ *H_x_* magnet. b) and e) Panels show the sweep‐rate dependent *M*(*H*) loops at a base temperature of 30 mK for **Ni_2_Tb** and **Ni_2_Ho**, respectively. c) and f) Panels contain the temperature‐dependent hysteresis loops with δ*H*/δ*t =* 16 mT/s. The data shown in panels (c–f) were collected with the magnetic field aligned along the easy axis of the crystal, as determined in the angular maps shown in (a and d).

We observe similar (but less pronounced) effects for the case of **Ni_2_Tb** and **Ni_2_Ho**, as the µSQUID studies (with field applied along the easy axis) also reveal open hysteresis loops (Figure 4). For **Ni_2_Tb**, the loops relax sharply near zero field with an additional step around ±0.3 T. These loops remain hysteretic up to 0.3 K, indicating a smaller magnetic anisotropy compared to **Ni_2_Dy** (Figure 3a–c). In the case of **Ni_2_Ho**, the loops display a slightly wider opening, with a rapid transition at ±0.1 T, and show hysteresis up to 0.2 K (Figure 3d–f).

Expectedly, no hysteresis is observed for **Zn_2_Tb**, indicating that the presence of Ni^2+^ is essential for enhancing the magnetic properties of the Tb^3+^ center. Due to the lack of crystals, µSQUID measurements could not be performed for **Zn_2_Ho**. However, based on the CASSCF results for Ho^3+^, no slow relaxation behavior is expected for this system either. Furthermore, µSQUID investigations of the **Ni_2_Er** and **Ni_2_Yb** analogs reveal closed loops (See Figure S8), i.e., 3d‐4f interactions are unable to uplift these systems, possibly due to the prolate orbitals and very fast relaxation dynamics leading to the absence of SMM behavior in these systems.

The easy axis in these systems was found to lie exactly perpendicular to the hexagonal‐shaped plane of the crystal. The drastic enhancement in the special **Ni_2_Ln** compounds compared to their “3d or 4f only” counterparts, the well‐defined easy axis, and the slow relaxation achieved by optimal use of 3d‐4f coordination in such systems open avenues for their technological applications, such as molecular quantum memory devices, where fast relaxation hampers the memory efficiency. This motivates us to thoroughly understand these systems via investigating the underlying Zeeman diagrams.

### Theoretical Insight

Our µSQUID investigations reveal that only the **Ni_2_Dy**, **Ni_2_Tb**, and **Ni_2_Ho** complexes exhibit slow relaxation behavior, in stark contrast to their not exchange‐coupled analogs—**Zn_2_Dy,** and **Zn_2_Tb**— and the weakly coupled **Ni_2_Y** counterpart, which show a faintly mild or no such characteristics. Similarly, no slow relaxation is observed for the **Ni_2_Er** and **Ni_2_Yb** systems. To gain deeper insight into the electronic structure and magnetic behavior of the **Ni_2_Ln** complexes, we consider a spin Hamiltonian of the following form:

(2) $$\begin{array}{cc} {\hat{H}}_{\mathrm{Ni}-4f}\; & =\sum_{i=1}^{2}\left\{\mu_{B}B^{T}g_{i}{\hat{S}}_{i\mathrm{Ni}}+hD_{i}\left({\hat{S}}_{\mathrm{zi},\mathrm{Ni}}^{2}-S\left(S+1\right)/3\right)\right.\\ & \;\left.+\;hE_{i}\left({\hat{S}}_{\mathrm{xi},\mathrm{Ni}}^{2}-{\hat{S}}_{\mathrm{yi},\mathrm{Ni}}^{2}\right)\right\}+\mu_{B}B^{T}g_{J}{\hat{J}}_{\mathrm{Ln}}\\ & \;+\sum_{k=2,4,6,-k\leq q\leq k}\;B_{q}^{k}O_{q}^{k}-h2J_{\mathrm{Ni}-\mathrm{Ni}}{\hat{S}}_{\mathrm{Ni}1}^{T}\cdot {\hat{S}}_{\mathrm{Ni}2}-h2J_{\mathrm{Ni}-\mathrm{Ln}}\\ & \left({\hat{S}}_{\mathrm{Ni}1}^{T}\cdot {\hat{S}}_{\mathrm{Ln}}+{\hat{S}}_{\mathrm{Ni}2}^{T}\cdot {\hat{S}}_{\mathrm{Ln}}\right) \end{array}$$

where the first term is the Zeeman energy, the second and third terms are the ZFS and Rhombic terms of the Ni^2+^ ions. The fourth and fifth terms are the Zeeman and ligand field Hamiltonian of the Ln^3+^ centers expressed as Steven's operators. The sixth term is the interaction operating between the Ni^2+^ ions, whilst the last term corresponds to the interaction between the Ni^2+^ and the Ln^3+^ centers.

By employing Hamiltonian (2), it is possible to quantify the strength of the magnetic interactions between the Ni^2+^ and Ln^3+^ centers. This was achieved by simultaneously fitting χ_M_ *T*(*T*) and *M*(*H*), data to Equation (2), using ligand field parameters for the lanthanides obtained from CASSCF calculations. To isolate the Ni^2+^–Ni^2+^ interaction, the **Ni_2_Y** complex was used as a reference. The fitting yielded a zero‐field splitting parameter of *D* = −1.6(2) cm^−1^ and an exchange coupling constant *J*_Ni–Ni_ = −0.106(1) cm^−1^ (Table S20). The ZFS value is consistent with that obtained from CASSCF, confirming the reliability of the computational approach.

For the lanthanide‐containing complexes, only the *g*‐values and ***J***_Ni‐Ni_ and ***J***_Ni‐Gd_ exchange interactions involving Ni^2+^ were fitted, while all other parameters were fixed to those derived from CASSCF calculations. The fits confirm the presence of ferromagnetic Ni···Ln interactions in the **Ni_2_Tb**, **Ni_2_Dy,** and **Ni_2_Ho** complexes, while antiferromagnetic coupling is observed for **Ni_2_Er** and **Ni_2_Yb** (Figure 2a–c). Notably, ferromagnetic interactions are only found in systems that exhibit open hysteresis loops, reinforcing the correlation between magnetic coupling and slow relaxation behavior (Table S20). The ***J***_Ni‐Ni_ interaction is found to be negative for **Ni_2_Tb** and **Ni_2_Dy,** while for the remaining systems it becomes ferromagnetic.

To assess the nature of these interactions—whether they arise purely from dipolar origin or also involve exchange contributions—we estimate the dipolar interaction strength between the Ln^3+^ and the Ni^2+^ ions (*J*_dip_). This calculation employs the *g*‐values obtained from CASSCF, the experimentally determined Ni···Ni and Ni···Ln distances, and Equation (3):

(3) $$J_{\text{dip}}=\frac{\mu_{0}\mu_{B}^{2}}{4r^{3}}\left[{\bar{\bar{g}}}_{A}.{\bar{\bar{g}}}_{B}-3\left({\bar{\bar{g}}}_{A}.\overset{⇀}{R}\right).\left({\overset{⇀}{R}}^{T}.{\bar{\bar{g}}}_{B}\right)\right]$$

where µ_B_ is the Bohr magneton, *r* is the Ln⋅⋅⋅Ni distance obtained from single‐crystal X‐ray analysis. ${\bar{\bar{g}}}_{Ln}$ is the *g*‐matrix for the ions, and $\overset{⇀}{R}$ is the directional unit vector connecting the ions (0 0 1). Due to the Ising nature of the ground states for **Ni_2_Dy**, **Ni_2_Tb,** and **Ni_2_Ho,** solely the ${\bar{\bar{g}}}_{zz}$ component of the ${\bar{\bar{g}}}_{Ln}$ matrix is non‐zero, i.e., ${\bar{\bar{g}}}_{\mathrm{zz}}$ ∼ 20 for Dy^3+^, ${\bar{\bar{g}}}_{\mathrm{zz}}$ ∼ 18 for Tb^3+^, and ${\bar{\bar{g}}}_{\mathrm{zz}}$ ∼ 16 for Ho^3+^. In such a scenario, the dipolar interaction *J*_dip_ is found to be +0.039 cm^−1^ for **Ni_2_Dy** (*J* = 15/2), +0.048 cm^−1^ for **Ni_2_Tb** (*J* = 6), and +0.034 cm^−1^ for **Ni_2_Ho** (*J* = 7). Comparison between *J*_dip_ and the experimentally determined *J*_ex_ reveals clear discrepancies, indicating that the magnetic interactions in these systems are not purely dipolar in nature. Specifically, the fitted parameters suggest the presence of exchange interactions of ferromagnetic origin in the **Ni_2_Tb**, **Ni_2_Dy**, and **Ni_2_Ho** complexes.

**Figure 5:**
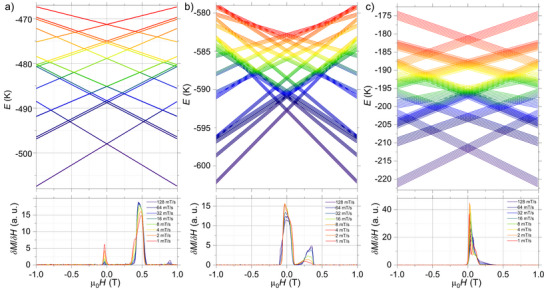
Zeeman diagrams for a) **Ni_2_Dy**, b) **Ni_2_Tb**, and c) **Ni_2_Ho** and the derivative of the *M*(*H*) loops (bottom panels).

Further insight into the magnetic behavior of these systems can be gained by comparing their Zeeman diagrams with the corresponding µSQUID hysteresis loops (Figure 5). For **Ni_2_Dy**, the loops are notably wide at 30 mK, featuring distinct magnetization jumps at 0 T, ∼0.5 T, and ∼0.88 T (Figure 5a). The small jump at zero field is attributed to a QTM, involving a three‐body co‐tunneling process, which occurs with significantly lower probability compared to two‐body and one‐body tunneling processes.^[^ ^21^, ^22^, ^48^ ^]^ The transition at ∼0.5 T, on the other hand, consists of a few overlapping crossings, some of which are one or two‐body tunneling with larger probability, as evidenced by derivative *M*(*H*). The final event at ∼0.88 T involves a two‐body tunneling process. Comparing the Zeeman diagrams between **Ni_2_Y**, **Zn_2_Dy,** and **Ni_2_Dy** side‐by‐side (see Figure S8), it is evident that shifting one, two‐body tunneling away from the zero field, as well as the ground to excited states separation, is important to yield large open loops. Moreover, it is clear from the Zeeman diagrams of **Ni_2_Dy** that although some excited states are closer than the single metal cases, and a zero‐field three‐body tunneling path exists, the zero‐field QTM is strongly quenched, allowing the large hysteresis to appear. Note that such observed effects are a direct result of exchange bias fields, observed before, often in dimeric^[^ ^13^, ^49^ ^]^ or trimer systems.^[^ ^16^ ^]^ The negligible transverse ligand field parameters, all‐axial alignments, and dominance of interactions in the axial direction make the **Ni_2_Ln** (**Tb**, **Dy,** and **Ho**) systems appealing and highlight key aspects for the optimum outcome from multi‐spin and particularly 3d‐4f coordination systems.

In the **Ni_2_Tb** and **Ni_2_Ho** systems, the sharp jump at zero field is similarly assigned to QTM at the zero‐field crossing. However, in addition, the broader hysteresis loops observed in these systems may be influenced by nuclear spin (hyperfine) effects. This is exemplified in the Zeeman diagrams for **Ni_2_Tb** and **Ni_2_Ho**, which include nuclear spin and hyperfine interactions.^[^ ^50^, ^51^ ^]^ In **Ni_2_Tb**, the hyperfine coupling splits the zero‐field QTM crossing across a field range of ±0.060 T (Figure 5b). Experimentally, the transition occurs over ∼0.1 T, likely due to dipolar broadening. A second crossing arises from QTM at excited‐state level crossings due to the remaining population in the excited state. In **Ni_2_Ho**, a single broadened crossing is observed, consistent with hyperfine interactions involving the Ho^3+^ nuclear spin (Figure 5c).

Finally, although several 3d–4f complexes with similar metallic cores to those presented here have been reported, the combination of low blocking temperatures, multiple molecular orientations, and/or transition metals with lower axiality makes a direct comparison of 3d–4f effects above 1 K challenging.^[^ ^27^, ^28^, ^30^, ^31^, ^32^, ^33^, ^34^, ^35^, ^36^, ^37^ ^]^ It is therefore possible that such effects are present but remain obscured under these conditions. In contrast, our investigation targets the milli‐kelvin regime, where these interactions become dominant, and the negligible transverse terms in the Hamiltonian enable a clear and direct assessment of the 3d–4f contribution.

## Conclusion

This study demonstrates that **Ni_2_Ln** complexes with linear 3d–4f–3d architectures can act as molecular quantum analogs of classical exchange spring magnets, bridging the gap between macroscopic and molecular‐scale magnetic phenomena. Through a detailed analysis, we reveal that the strategic pairing of a reduced “hard” magnetic phase (Ni^2+^) with a “soft” lanthanide center (Ln^3+^) fosters ferromagnetic 3d–4f coupling while minimizing transverse interactions, thereby enhancing magnetic bistability and supporting slow relaxation dynamics in selected systems.

Specifically, **Ni_2_Tb**, **Ni_2_Dy**, and **Ni_2_Ho** exhibit open hysteresis loops and slow magnetic relaxation, indicative of robust SMM behavior in agreement with the CASSCF‐predicted axial anisotropies and strong exchange interactions. In contrast, complexes incorporating diamagnetic or weakly anisotropic components such as **Ni_2_Y, Zn_2_Tb/Dy**, **Ni_2_Er**, and **Ni_2_Yb** fail to exhibit SMM characteristics, highlighting the critical interplay between electronic structure, axiality, and exchange strength in governing the magnetic properties.

The analogy of molecular exchange spring magnets deepens our understanding of intramolecular magnetic cooperativity and offers a versatile design strategy for engineering next‐generation magnetic molecular units. These findings underscore the potential of 3d‐4f heterometallic systems as tunable platforms for advanced magnetic materials, with promising applications in high‐density data storage, spintronics, and quantum technologies where precise control over magnetic relaxation and bistability is essential.

## Conflict of Interests

The authors declare no conflict of interest.

## Supporting information



Supporting information

## Data Availability

The data that support the findings of this study are available from the corresponding author upon reasonable request.
