# Supplementary material for: Leveraging 3d‐4f Coordination: Molecular Quantum Spring‐Magnet Behavior in Axial Ni2Ln Complexes
Source: Angew Chem Int Ed Engl. 2025 Dec 19;65(5):e22076. doi: 10.1002/anie.202522076 (PMC12851015; doi:10.1002/anie.202522076)
Supplement: Supplementary file 1 — Supporting information [file ANIE-65-e22076-s001.docx]

***Supplementary Information***

***Leveraging 3d-4f Coordination: Molecular Quantum Spring-Magnet Behavior in Axial Ni_2_Ln Complexes***

Zhaoyang Jing*^a^*, Eufemio Moreno-Pineda*^b,c,d*^*, Sagar Paul*^b*^*, Appu Sunil*^b^*, Olaf Fuhr*^a,e^*, Yaorong Chen^a^, Wolfgang Wernsdorfer*^b,f*^*, and Mario Ruben*^a,f,g*^*

*^a^ Institute of Nanotechnology (INT), Karlsruhe Institute of Technology (KIT), Hermann-von-Helmholtz-Platz 1, D-76344, Eggenstein-Leopoldshafen, Germany.*

*^b^ Physikalisches Institut, Karlsruhe Institute of Technology, D-76131 Karlsruhe, Germany.*

*^c^ Universidad de Panamá, Facultad de Ciencias Naturales, Exactas y Tecnología, Depto. de Química-Física, 0824 Panamá, Panamá.*

*^d^ Universidad de Panamá, Facultad de Ciencias Naturales, Exactas y Tecnología, Grupo de Investigación de Materiales, 0824 Panamá, Panamá.*

*^e^ Karlsruhe Nano Micro Facility (KNMFi), Karlsruher Institut für Technologie (KIT), Kaiserstraße 12, 76131 Karlsruhe, Germany*

*^f^ Institute of Quantum Materials and Technologies (IQMT), Karlsruhe Institute of Technology (KIT), Hermann-von-Helmholtz-Platz 1, D-76344, Eggenstein-Leopoldshafen, Germany.*

*^g^ Centre Européen de Sciences Quantiques (CESQ), Institut de Science et d’Ingénierie Supramoléculaires (ISIS), 8 allée Gaspard Monge, BP 70028, 67083, Strasbourg Cedex, France.*

*Correspondence to: [eufemio.moreno@up.ac.pa](mailto:eufemio.moreno@up.ac.pa); [sagar.paul@kit.edu](mailto:sagar.paul@kit.edu); [wolfgang.wernsdorfer@kit.edu](mailto:wolfgang.wernsdorfer@kit.edu); [mario.ruben@kit.edu](mailto:mario.ruben@kit.edu)

1. **Experimental Section**
   1. **Synthetical Details**

**Tris(((2-hydroxy-3-methoxybenzyl)-amino)ethyl)amine (H_3_L)**: Three equivalents of *O*-vanillin (2.28 g, 15.0 mmol) were added to a clear colourless solution of tris(2-aminoethyl)amine (0.73g, 5.0 mmol) in MeOH (15 mL) at room temperature. The resulting yellow solution was stirred for 4 hours. During the stirring, thick yellow precipitate started to form. The precipitate was filtered, washed with cold MeOH and diethyl ether and dried in an oven at 60 °C overnight to give 2.12 g H_3_L (C_30_H_36_O_6_N_4_, FW = 548.63, yield 77.1%).

**Nickel(II) trifluoromethanesulfonate (Ni(CF_3_SO_3_)_2_)**: Slightly excessive trifluoromethanesulfonic acid (1.80 g, 12.0 mmol) was added dropwise to a clear green solution of Ni(OAc)_2_·4H_2_O (1.24 g, 5.0 mmol) in MeCN (100 mL) at room temperature. After stirring for 1 hour, the mixture was concentrated under reduced pressure to around 15 mL, and diethyl ether (100 mL) was added. The resulting blue precipitation was then filtered and washed with cold diethyl ether and hexane. After drying in an oven at 60 °C overnight, 1.12g Ni(CF_3_SO_3_)_2_ (FW = 548.63, yield 62.7 %) was obtained.

**[Ln(NiL)_2_]CF_3_SO_3_ (Ni_2_Ln, Ln = Tb^3+^, Dy^3+^, Ho^3+^, Er^3+^, Yb^3+^, Y^3+^)**: H_3_L (27.5 mg, 0.05 mmol) and Ni(CF_3_SO_3_)_2_ (18.0 mg, 0.05 mmol) was dissolved together in 12 mL methanol. The solution was first stirred for 10 mins after adding triethylamine (30.0 mg, 0.30 mmol), and then for another 10 mins upon the addition of 0.025 mmol Ln(CF_3_SO_3_)_3_. Slow evaporation of the final yellow solution at room temperature gave green crystals of hexagonal prism shape after 2 to 3 days (yield 24.1**–**31.2%). Anal. Calcd. for **Ni_2_Tb** (TbNi_2_C_61_H_66_F_3_N_8_O_15_S, FW = 1516.61 g·mol^−1^): C, 48.31; H, 4.39; N, 7.39. Found: C, 48.23; H, 4.17; N, 7.40. Anal. Calcd. for **Ni_2_Dy** (DyNi_2_C_61_H_66_F_3_N_8_O_15_S, FW = 1520.19 g·mol^−1^): C, 48.20; H, 4.38; N, 7.37. Found: C, 48.25; H, 4.33; N, 7.39. Anal. Calcd. for **Ni_2_Ho** (HoNi_2_C_61_H_66_F_3_N_8_O_15_S, FW = 1522.59 g·mol^−1^): C, 48.12; H, 4.37; N, 7.36. Found: C, 48.01; H, 4.22; N, 7.24. Anal. Calcd. for **Ni_2_Er** (ErNi_2_C_61_H_66_F_3_N_8_O_15_S, FW = 1524.95 g·mol^−1^): C, 48.04; H, 4.36; N, 7.35. Found: C, 47.76; H, 4.28; N, 7.26. Anal. Calcd. for **Ni_2_Yb** (YbNi_2_C_61_H_66_F_3_N_8_O_15_S, FW = 1530.73 g·mol^−1^): C, 47.86; H, 4.35; N, 7.32. Found: C, 47.43; H, 4.25; N, 7.18. Anal. Calcd. for **Ni_2_Y** (YNi_2_C_61_H_66_F_3_N_8_O_15_S, FW = 1446.60 g·mol^−1^): C, 50.64; H, 4.60; N, 7.75. Found: C, 50.24; H, 4.42; N, 7.63.

**[Ln(ZnL)_2_]CF_3_SO_3_ (Zn_2_Ln, Ln = Tb, Dy)**:

**Step 1:** H_3_L (1.1 g, 2.0 mmol) and Zn(CH_3_COO)_2_ (0.37 g, 2.0 mmol) was dissolved in 20 mL of methanol. The solution was evaporated to dryness, and the resulting orange oil was dissolved in 20 mL of methanol and dried again. This procedure was repeated 5 times to remove the acetate anions. The resulting oil is then used directly in the next step.

**Step 2:** The Oily product (66.0 mg) prepared from the first step was dissolved in 4 mL methanol together with Dy(CF_3_SO_3_)_3_ (30.5 mg, 0.05 mmol). Then, a 1 mL ethanol solution containing triethylamine (60.0 mg, 0.6 mmol) was added to the former methanol solution. The mixture solution was stirred for 5 mins and left to stand in an incubator at 45℃. Yellow hexagonal prismatic crystals were obtained after 2 days (yield 35.4%). Anal. Calcd for **Zn_2_Tb** (TbZn_2_C_61_H_66_F_3_N_8_O_15_S, FW = 1529.93 g·mol^−1^): C, 47.89; H, 4.35; N, 7.32. Found: C, 48.13; H, 4.29; N, 7.23. Anal. Calcd. for **Zn_2_Dy** (DyZn_2_C_61_H_66_F_3_N_8_O_15_S, FW = 1533.56 g·mol^−1^): C, 47.78; H, 4.34; N, 7.30. Found: C, 47.65; H, 4.22; N, 7.19.

**X-Ray Structure Determination**

Single crystal X-ray data of the complexes were collected at 180 K either on a STOE StadiVadi diffractometer with monochromated GaKα-radiation (λ_GaKα_ = 1.34143 Å) or on a STOE IPDS II diffractometer with monochromated MoKα-radiation (λ_MoKα_ = 0.71073 Å). The structure was solved by intrinsic phasing methods and refined with full-matrix least squares procedures on *F*2 using SHELXL 2018/3^1^ implemented in Olex2 1.5.^2^ The locations of the non-hydrogen atoms were determined from the difference Fourier maps. The non-hydrogen atoms were refined anisotropically, and all hydrogen atoms were introduced in calculated positions and refined with fixed geometry with respect to their carrier atoms. Crystallographic details are listed in Tables S1–S4. PXRD data were collected with a STOE STADI P diffractometer equipped with CuKα1 radiation at room temperature. Full crystallographic details can be found in CIF format: CCDC 2490677 (for **Ni_2_Y**), 2490678 (for **Ni_2_Tb**), 2490679 (for **Ni_2_Dy**), 2490680 (for **Ni_2_Ho**), 2490681 (for **Ni_2_Er**), 2490682 (for **Ni_2_Yb**), 2490683 (for **Zn_2_Dy**), 2490684 (for **Zn_2_Tb**). These data are provided free of charge by the joint Cambridge Crystallographic Data Centre and Fachinformationszentrum Karlsruhe via [www.ccdc.cam.ac.uk](https://www.ccdc.cam.ac.uk/).

- 1. **ATR-IR**

ATR-IR spectra were obtained using a Nicolet iS50 FTIR spectrometer, covering a range of 400–4000 cm^−1^.

## Magnetic Measurements

Magnetic susceptibility was measured on polycrystalline samples using a Quantum Design MPMS-XL SQUID magnetometer equipped with a 1 T magnet, in the temperature range of 2.0 to 300 K under a dc magnetic field of 1 kOe. The ac data was collected using an oscillating magnetic field of 3.5 Oe and frequencies between 1 Hz and 1.5 kHz under zero dc field. Magnetisation data were collected with a Quantum Design MPMS3 SQUID magnetometer equipped with a 7 T magnet. The samples were ground and fixed in gelatine capsules using small amounts of eicosane to avoid any movement of the samples. The data obtained were corrected for diamagnetic contributions of the eicosane, the gelatine capsule and the sample holder. Diamagnetic corrections for the sample were estimated using Pascal’s constants. Low temperature (0.03 – 5 K) magnetization measurements were performed on single crystals using a μSQUID apparatus. In all cases, the field was aligned along the easy axis of the crystal, employing the transverse field method. The studies were carried out on single crystals with the field applied along the principal anisotropic axis. Hysteresis loops were obtained at different sweep rates and temperatures, ranging from 5 K to 30 mK and from 1 to 128 mT/s.

## Computational Details

Complete-active-space self-consistent field (CASSCF) calculations on the basis of single-crystal X-ray determined geometry have been carried out with OpenMolcas program package. For the **Ni_2_Ln** systems, each Ln^3+^ complex was calculated keeping the experimentally determined structure of the corresponding compound, while one Ln^3+^ was replaced for Y^3+^ and the Ni^2+^ ions were replaced by diamagnetic Zn^2+^. To investigate the Ni^2+^ ion in each complex, the Ln^3+^ ions were replaced by diamagnetic Y^3+^. The basis sets for all atoms are atomic natural orbitals from the MOLCAS ANO-RCC library: ANO-RCC-VTZP for Ln^3+^ and Ni^2+^; VDZP for close N and O; VDZ for distant atoms. The calculations employed the second-order Douglas-Kroll-Hess Hamiltonian, where scalar relativistic contractions were taken into account in the basis set and the spin-orbit couplings were handled separately in the restricted active space state interaction (RASSI-SO) procedure. For the individual Ln^3+^ fragment, active electrons in 7 active spaces include all *f* electrons in the CASSCF calculation. For the individual Ni^2+^ fragment, the active electrons in the 5 active spaces include all *d* electrons in the CASSCF calculation. We calculated all the roots in the active space. For the individual Ni^2+^ fragment, all *d* electrons (CAS (8 in 5)) and all 10 triplets, 15 singlets are included in the calculation. SINGLE_ANISO program was used to obtain the energy levels, ***g*** tensors, *m_J_* values, magnetic axes, *et al.,* based on the above CASSCF/RASSI-SO calculations.

1. **Crystallographic Tables and Figures**

**Table S1.** Crystallographic information data for **Ni_2_Tb**, **Ni_2_Dy** and **Ni_2_Ho**.

|  | **Ni_2_Tb** | **Ni_2_Dy** | **Ni_2_Ho** |
| --- | --- | --- | --- |
| Formula | TbC_61_H_66_F_3_N_8_Ni_2_O_15_S | DyC_61_H_66_F_3_N_8_Ni_2_O_15_S | HoC_61_H_66_F_3_N_8_Ni_2_O_15_S |
| Fw | 1516.61 | 1520.19 | 1522.59 |
| Temp (K) | 180.0 | 180.0 | 180.0 |
| Radiation | MoKα | GaKα | MoKα |
| Crystal system | trigonal | trigonal | trigonal |
| Space group | *R*32 | *R*32 | *R*32 |
| *a* (Å) | 11.6865(6) | 11.8738(4) | 11.9220(4) |
| *b* (Å) | 11.6865(6) | 11.8738(4) | 11.9220(4) |
| *c* (Å) | 38.324(2) | 38.4123(15) | 38.1382(14) |
| α (°) | 90 | 90 | 90 |
| β (°) | 90 | 90 | 90 |
| γ (°) | 120 | 120 | 120 |
| Volume (Å^3^) | 4532.8(5) | 4690.1(4) | 4694.5(4) |
| *Z* | 3 | 3 | 3 |
| *D*_calc_ (g cm^-3^) | 1.667 | 1.615 | 1.616 |
| *μ* (mm^-1^) | 1.894 | 10.034 | 1.963 |
| *F* (000) | 2316.0 | 2319.0 | 2322.0 |
| *R* _int_ | 0.0596 | 0.0494 | 0.0474 |
| *R*_sigma_ | 0.0314 | 0.0308 | 0.0221 |
| Refl. (all) | 15883 | 19127 | 20981 |
| Refl (independent) | 2322 | 2541 | 3179 |
| *R*_1_ (all) | 0.0565 | 0.0435 | 0.0505 |
| *R*_1_ (> 2σ) | 0.0457 | 0.0394 | 0.0447 |
| w*R*_2_ (all) | 0.1190 | 0.0988 | 0.1169 |
| w*R*_2_ (> 2σ) | 0.1129 | 0.0968 | 0.1127 |
| Flack parameter | 0.43(3) | 0.493(7) | 0.49(2) |
| GOF. | 1.049 | 0.994 | 1.078 |
| CCDC number | 2490678 | 2490679 | 2490680 |

$R_{1}=\sum\left| \left| F_{o} \right|-\left| F_{c} \right| \right|/\sum\left| F_{o} \right|$, $wR_{2}=\left[ \sum w\left( F_{o}^{2}-F_{c}^{2} \right)^{2}/\sum w\left( F_{o}^{2} \right)^{2} \right]^{1/2}$

**Table S2.** Crystallographic information data for **Ni_2_Er**, **Ni_2_Yb** and **Ni_2_Y**.

|  | **Ni_2_Er** | **Ni_2_Yb** | **Ni_2_Y** |
| --- | --- | --- | --- |
| Formula | ErC_61_H_66_F_3_N_8_Ni_2_O_15_S | YbC_61_H_66_F_3_N_8_Ni_2_O_15_S | YC_61_H_66_F_3_N_8_Ni_2_O_15_S |
| Fw | 1524.95 | 1530.73 | 1446.60 |
| Temp (K) | 180.0 | 180.0 | 180.0 |
| Radiation | MoKα | GaKα | MoKα |
| Crystal system | trigonal | trigonal | trigonal |
| Space group | *R*32 | *R*32 | *R*32 |
| *a* (Å) | 11.9585(3) | 12.0330(5) | 11.9363(4) |
| *b* (Å) | 11.9585(3) | 12.0330(5) | 11.9363(4) |
| *c* (Å) | 37.8448(11) | 37.2737(19) | 38.0756(18) |
| α (°) | 90 | 90 | 90 |
| β (°) | 90 | 90 | 90 |
| γ (°) | 120 | 120 | 120 |
| Volume (Å^3^) | 4686.9(3) | 4673.9(5) | 4698.0(4) |
| *Z* | 3 | 3 | 3 |
| *D*_calc_ (g cm^-3^) | 1.621 | 1.632 | 1.534 |
| *μ* (mm^-1^) | 2.043 | 2.202 | 1.629 |
| *F* (000) | 2325.0 | 2331.0 | 2238.0 |
| *R* _int_ | 0.0549 | 0.0989 | 0.2492 |
| *R*_sigma_ | 0.0272 | 0.0997 | 0.1089 |
| Refl. (all) | 20777 | 9060 | 23010 |
| Refl (independent) | 3188 | 3163 | 3605 |
| *R*_1_ (all) | 0.0582 | 0.1091 | 0.1270 |
| *R*_1_ (> 2σ) | 0.0470 | 0.0562 | 0.0889 |
| w*R*_2_ (all) | 0.1230 | 0.1371 | 0.2639 |
| w*R*_2_ (> 2σ) | 0.1160 | 0.1193 | 0.2259 |
| Flack parameter | 0.48(2) | 0.47(3) | 0.45(3) |
| GOF. | 1.069 | 0.962 | 1.039 |
| CCDC number | 2490681 | 2490682 | 2490677 |

$R_{1}=\sum\left| \left| F_{o} \right|-\left| F_{c} \right| \right|/\sum\left| F_{o} \right|$, $wR_{2}=\left[ \sum w\left( F_{o}^{2}-F_{c}^{2} \right)^{2}/\sum w\left( F_{o}^{2} \right)^{2} \right]^{1/2}$

**Table S3.** Crystallographic information data for **Zn_2_Tb** and **Zn_2_Dy**.

|  | **Zn_2_Tb** | **Zn_2_Dy** |
| --- | --- | --- |
| Formula | C_61_H_66_TbF_3_N_8_Zn_2_O_15_S | C_61_H_66_DyF_3_N_8_Zn_2_O_15_S |
| Fw | 1529.93 | 1533.51 |
| Temp (K) | 180.0 | 180.0 |
| Radiation | MoKα | GaKα |
| Crystal system | trigonal | trigonal |
| Space group | *R*32 | *R*32 |
| *a* (Å) | 11.8932(4) | 11.9481(3) |
| *b* (Å) | 11.8932(4) | 11.9481(3) |
| *c* (Å) | 38.5749(16) | 38.4497(11) |
| α (°) | 90 | 90 |
| β (°) | 90 | 90 |
| γ (°) | 120 | 120 |
| Volume (Å^3^) | 4725.3(4) | 4753.6(3) |
| *Z* | 3 | 3 |
| *D*_calc_ (g cm^-3^) | 1.613 | 1.607 |
| *μ* (mm^-1^) | 1.980 | 7.268 |
| *F* (000) | 2328.0 | 2331.0 |
| *R* _int_ | 0.0519 | 0.0453 |
| *R*_sigma_ | 0.0485 | 0.0150 |
| Refl. (all) | 14703 | 30498 |
| Refl (independent) | 3259 | 30498 |
| *R*_1_ (all) | 0.0428 | 0.312 |
| *R*_1_ (> 2σ) | 0.0329 | 0.311 |
| w*R*_2_ (all) | 0.0747 | 0.0847 |
| w*R*_2_ (> 2σ) | 0.0733 | 0.0845 |
| Flack parameter | -0.022(16) | 0.390(5) |
| GOF. | 0.999 | 1.068 |
| CCDC number | 2490684 | 2490683 |

$R_{1}=\sum\left| \left| F_{o} \right|-\left| F_{c} \right| \right|/\sum\left| F_{o} \right|$, $wR_{2}=\left[ \sum w\left( F_{o}^{2}-F_{c}^{2} \right)^{2}/\sum w\left( F_{o}^{2} \right)^{2} \right]^{1/2}$

# **Table S4.** Selected bond lengths (Å) and bond angles (°).

|  | **Ni_2_Tb** | **Ni_2_Dy** | **Ni_2_Ho** | **Ni_2_Er** | **Ni_2_Yb** | **Ni_2_Y** |
| --- | --- | --- | --- | --- | --- | --- |
| Ln1···Ni1 | 3.2501(10) | 3.2442(8) | 3.2134(8) | 3.1815(8) | 3.1211(12) | 3.2058(13) |
| Ln1–O1 | 2.384(4) | 2.346(3) | 2.326(3) | 2.307(4) | 2.268(5) | 2.323(5) |
| Ni1–O1 | 2.036(4) | 2.062(3) | 2.065(3) | 2.064(4) | 2.071(5) | 2.065(5) |
| Ni1–N1 | 2.058(5) | 2.089(7) | 2.094(9) | 2.086(5) | 2.074(6) | 2.083(6) |
| ∠Ni1–Ln1–Ni1^1^ | 180.0 | 180.0 | 180.0 | 180.0 | 180.0 | 180.0 |
| ∠O1–Ni1–O1^2^ | 77.0(2) | 77.3(1) | 77.4(2) | 77.6(2) | 78.0(2) | 77.6(2) |
| ∠ N2–Ni1–N2^2^ | 96.9(2) | 97.3(1) | 97.4(2) | 97.5(2) | 97.4(2) | 97.4(3) |
| ∠ Ln1–O1–Ni1 | 95.4(2) | 94.6(1) | 93.9(1) | 93.2(1) | 91.9(2) | 93.7(2) |
| ∠ O1–Ln1–O1^2^ | 65.3(2) | 66.5(1) | 67.5(1) | 68.2(1) | 70.1(2) | 67.7(2) |

Symmetry codes: ^1^-1/3+Y,1/3+X,-2/3-Z; ^2^-1+Y-X,-1-X,+Z.

**Table S5.** Selected bond lengths (Å) and bond angles (°).

|  | **Zn_2_Tb** | **Zn_2_Dy** |
| --- | --- | --- |
| Ln1···Zn1 | 3.3704(6) | 3.3493(8) |
| Ln1—O1 | 2.371(2) | 2.349(3) |
| Zn1—O1 | 2.144(2) | 2.155(3) |
| Zn1—N1 | 2.129(3) | 2.130(4) |
| ∠ Zn1—Ln1—Zn1^1^ | 180.0 | 180.0 |
| ∠ O1—Zn1—O1^2^ | 74.5(1) | 74.3(1) |
| ∠ N2—Zn1—N2^2^ | 100.8(1) | 100.9(1) |
| ∠ Ln1—O1—Zn1 | 96.45(8) | 96.0(1) |
| ∠ O1—Ln1—O1^2^ | 66.36(8) | 67.3(1) |

Symmetry codes: ^1^-1/3+Y,1/3+X,-2/3-Z; ^2^-1+Y-X,-1-X,+Z.

**Table S6.** Continuous Shape Measures for Ni^2+^.

| **Label** | **Shape** | **Ni_2_Tb** | **Ni_2_Dy** | **Ni_2_Ho** | **Ni_2_Er** | **Ni_2_Yb** | **Ni_2_Y** | **Zn_2_Tb** | **Zn_2_Dy** |
| --- | --- | --- | --- | --- | --- | --- | --- | --- | --- |
| HP-6 (*D*_6_*_h_*) | Hexagon | 33.085 | 33.136 | 33.285 | 33.282 | 33.225 | 33.224 | 33.998 | 33.920 |
| PPY-6 (*C*_5_*_v_*) | Pentagonal pyramid | 26.585 | 27.350 | 26.542 | 26.702 | 26.866 | 26.772 | 25.000 | 25.199 |
| OC-6 (*O_h_*) | Octahedron | **1.187** | **1.101** | **1.198** | **1.128** | **1.016** | **1.106** | **2.129** | **2.042** |
| TPR-6 (*D*_3_*_h_*) | Trigonal prism | 12.495 | 13.469 | 12.526 | 12.704 | 12.843 | 12.758 | 10.875 | 11.060 |
| JPPZ-6 (*C*_5_*_v_*) | Johnson pentagonal pyramid (J2) | 30.531 | 31.307 | 30.508 | 30.646 | 30.764 | 30.708 | 28.986 | 29.168 |

**Table S7.** Continuous Shape Measures for Ln^3+^.

| **Label** | **Shape** | **Ni_2_Tb** | **Ni_2_Dy** | **Ni_2_Ho** | **Ni_2_Er** | **Ni_2_Yb** | **Ni_2_Y** | **Zn_2_Tb** | **Zn_2_Dy** |
| --- | --- | --- | --- | --- | --- | --- | --- | --- | --- |
| HP-6 (*D*_6_*_h_*) | Hexagon | 29.291 | 29.128 | 29.391 | 29.440 | 29.663 | 29.453 | 29.924 | 29.982 |
| PPY-6 (*C*_5_*_v_*) | Pentagonal pyramid | 27.695 | 28.276 | 26.715 | 26.395 | 25.414 | 26.447 | 25.368 | 24.987 |
| OC-6 (*O_h_*) | Octahedron | **8.434** | **7.738** | **7.441** | **7.068** | **6.396** | **7.434** | 8.992 | 8.567 |
| TPR-6 (*D*_3_*_h_*) | Trigonal prism | 10.684 | 11.440 | 9.709 | 9.405 | 8.431 | 9.408 | **8.018** | **7.656** |
| JPPZ-6 (*C*_5_*_v_*) | Johnson pentagonal pyramid (J2) | 30.615 | 31.192 | 29.771 | 29.500 | 28.642 | 29.526 | 28.458 | 28.135 |


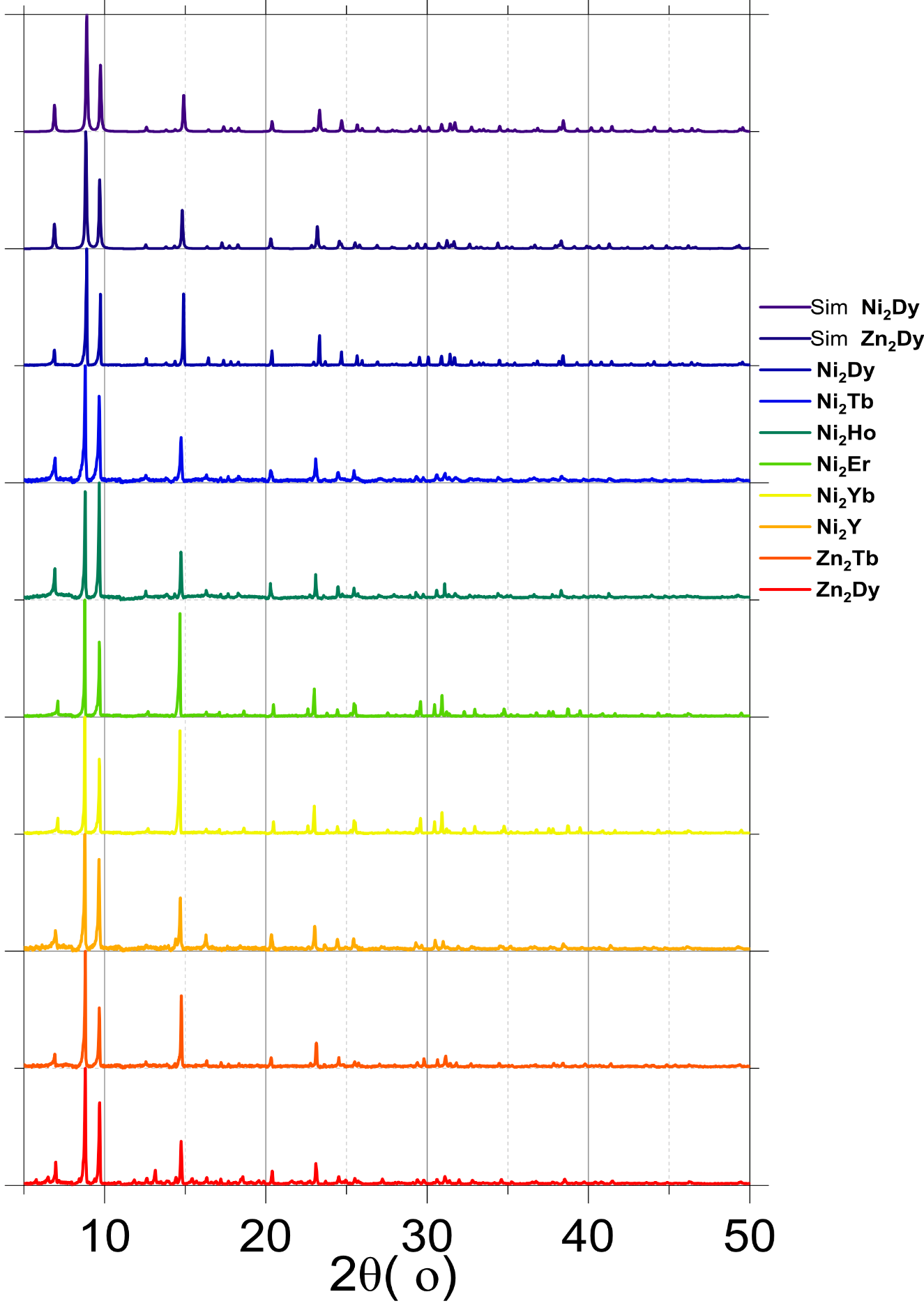


Figure S1. Experimental powder X-ray diffraction patterns of Ni_2_Ln and Zn_2_Ln complexes and simulated patterns for Ni_2_Dy and Zn_2_Dy.


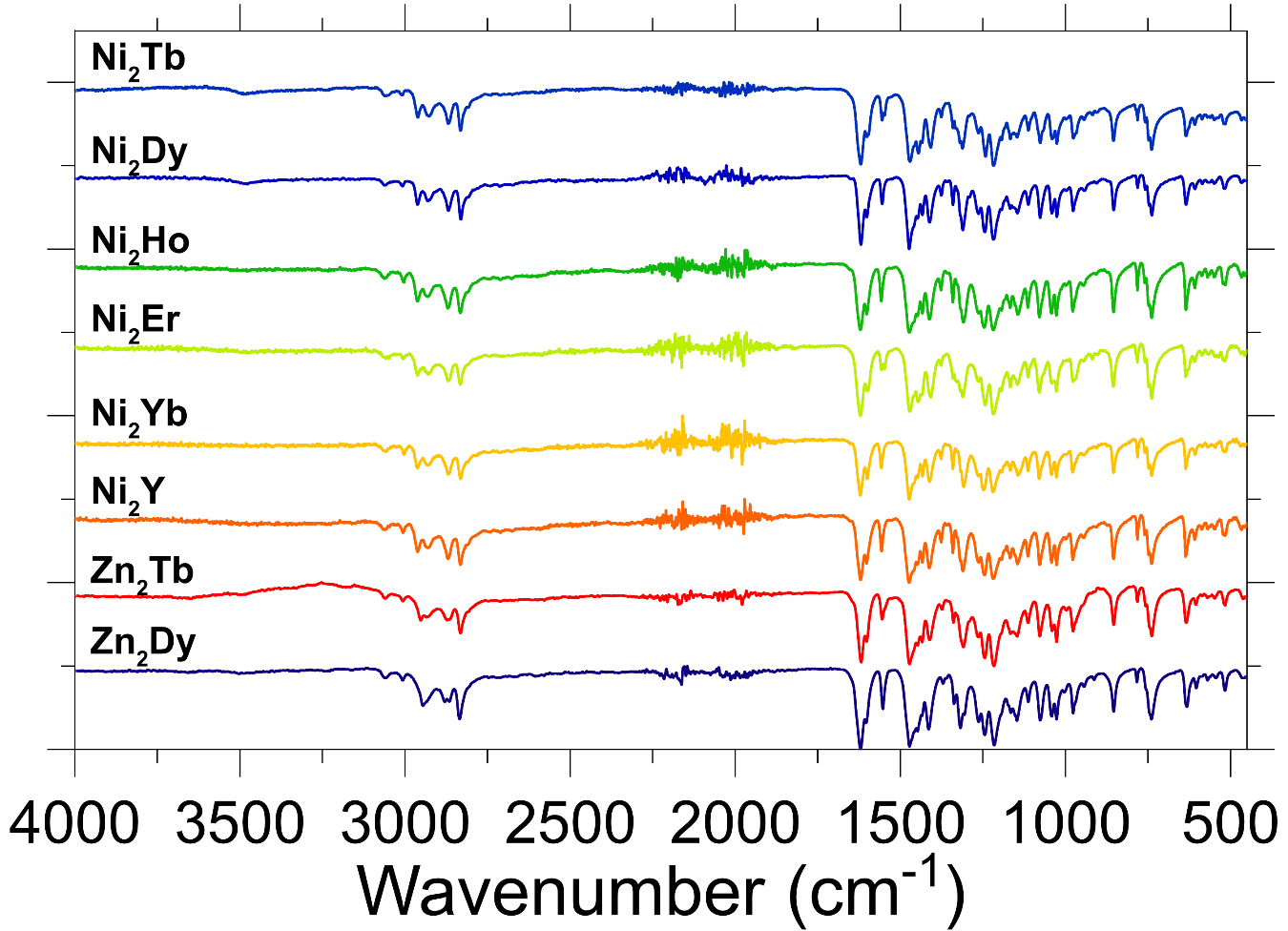


Figure S2. ATR Infrared studies for Ni_2_Ln and Zn_2_Ln complexes.


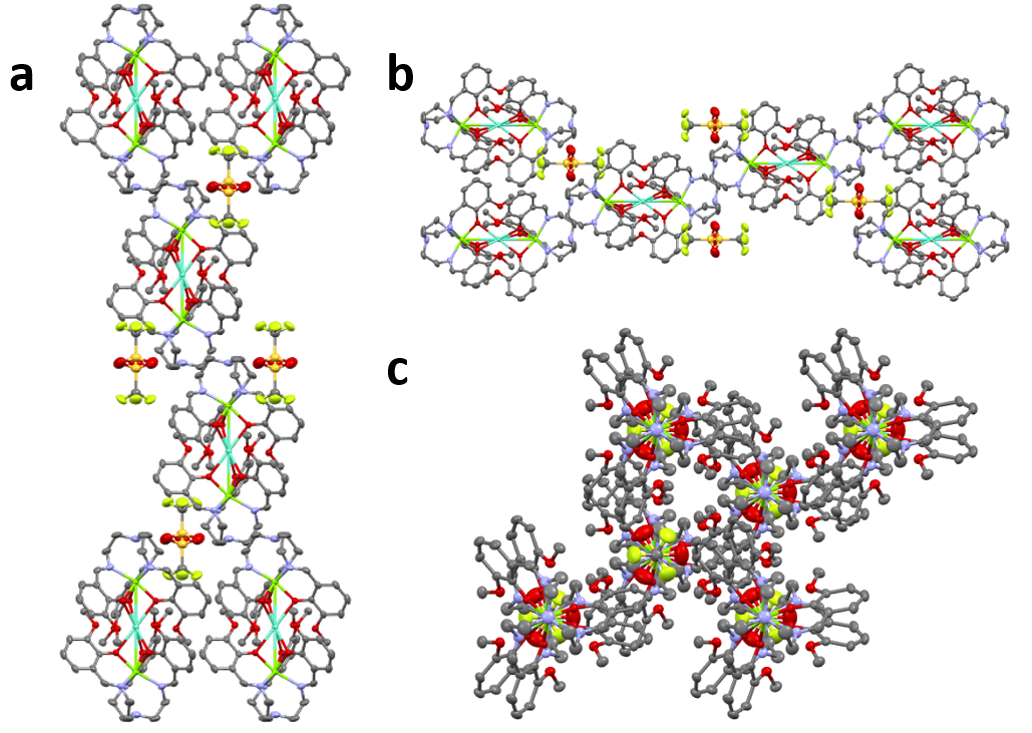


**Figure S3.** Packing diagram of **Ni_2_Dy** along the directions of *a*, *b*, *c* axes.

1. **Magnetic Tables and Figures**

**Table S8.** Room temperature and theoretical χ_M_*T* (cm^3^mol^−1^K) values for **Ni_2_Ln** and **Zn_2_Ln** series.

| Complexes | Free ion χ_M_*T* | Exp. χ_M_*T* (300 K) |
| --- | --- | --- |
| Ni_2_Y | 2.6 | 2.6 |
| Ni_2_Tb | 14.5 | 14.6 |
| Ni_2_Dy | 16.8 | 16.6 |
| Ni_2_Ho | 16.7 | 16.1 |
| Ni_2_Er | 14.1 | 14.2 |
| Ni_2_Yb | 5.21 | 5.1 |
| Zn_2_Tb | 11.8 | 11.6 |
| Zn_2_Dy | 14.2 | 14.1 |

- *The g value for the Ni^2+^ employed for the RT* χ_M_*T calculation was fixed to g*_Ni_ = 2.3**.**

**Table S9.** AC fitting parameters of the simultaneous fitting of the χ’_M_ and χ’’_M_ AC data.

| Temperature (K) | **χ**_T_  (cm^3^ mol^-1^) | **χ**_T (error)_ | **χ**_S_  (cm^3^ mol^-1^) | **χ**_S (error)_ | α | α error | τ (s) | +τ_error_ | -τ_error_ |
| --- | --- | --- | --- | --- | --- | --- | --- | --- | --- |
| 1.9 | 11.87 | 0.03 | 0.72 | 0.05 | 0.118 | 0.005 | 0.00121 | 0.0032 | 4.61108E-4 |
| 2.0 | 11.29 | 0.02 | 0.77 | 0.05 | 0.117 | 0.005 | 7.85114E-4 | 0.00201 | 3.0739E-4 |
| 2.1 | 10.78 | 0.02 | 0.84 | 0.06 | 0.105 | 0.005 | 5.2851E-4 | 0.00131 | 2.13673E-4 |
| 2.2 | 10.33 | 0.02 | 0.90 | 0.06 | 0.099 | 0.005 | 3.70305E-4 | 8.87323E-4 | 1.54539E-4 |
| 2.3 | 10.15 | 0.01 | 0.92 | 0.06 | 0.097 | 0.005 | 3.23452E-4 | 7.67461E-4 | 1.36321E-4 |
| 2.4 | 9.60 | 0.01 | 0.98 | 0.08 | 0.091 | 0.006 | 2.10062E-4 | 4.82093E-4 | 9.15306E-5 |
| 2.5 | 9.23 | 0.01 | 1.03 | 0.08 | 0.083 | 0.006 | 1.58829E-4 | 3.49704E-4 | 7.21373E-5 |
| 2.6 | 8.89 | 0.01 | 1.06 | 0.09 | 0.076 | 0.005 | 1.23465E-4 | 2.61375E-4 | 5.83208E-5 |

* The τ errors were determined via the distribution of α parameters.

**Table S10.** Calculated anisotropic parameters for Ni^2+^ in the different **Ni_2_Ln** hosts.

| **Ni_2_Ln** | ***D_X_*** / cm^-1^ | ***D_Y_*** / cm^-1^ | ***D_Z_*** / cm^-1^ | ***D*** / cm^-1^ | ***E*** / cm^-1^ | ***E*/*D*** | ***g*_x_** | ***g*_y_** | ***g*_z_** |
| --- | --- | --- | --- | --- | --- | --- | --- | --- | --- |
| **Y** | 0.879 | 0.880 | -1.759 | -2.6385 | -0.0005 | 0.000189 | 2.2895 | 2.2897 | 2.3003 |
| **Tb** | 0.407 | 0.408 | -0.815 | -1.2225 | -0.0005 | 0.000409 | 2.2689 | 2.2700 | 2.2724 |
| **Dy** | 0.697 | 0.698 | -1.395 | -2.0925 | -0.0005 | 0.000239 | 2.2928 | 2.2931 | 2.3008 |
| **Ho** | 0.834 | 0.838 | -1.672 | -2.508 | -0.002 | 0.000797 | 2.2851 | 2.2852 | 2.3054 |
| **Er** | 0.999 | 1.003 | -2.002 | -3.003 | -0.002 | 0.000666 | 2.2796 | 2.2796 | 2.3032 |
| **Yb** | 1.069 | 1.07 | -2.139 | -3.2085 | -0.0005 | 0.000156 | 2.2856 | 2.2859 | 2.2999 |

**Table S11.** Computed energy levels (the ground state is set at zero), wavefunction *m_J_* composition, of the ground-state multiplet ^7^F_6_ for the Tb^3+^ in **Ni_2_Tb**, at the CASSCF level.

|  | **Wavefunction** | | | | | |
| --- | --- | --- | --- | --- | --- | --- |
| *m_J_* | **State 1**  **(0 cm^-1^)** | **State 2**  **(0.018 cm^-1^)** | **State 3**  **(272.927 cm^-1^)** | **State 4**  **(272.9 27cm^-1^)** | **State 5**  **(464.915 cm^-1^)** | **State 6**  **(464.917 cm^-1^)** |
| $\left. \left\vert-6 \right. \right\rangle$ | 49.9 | 49.9 | 0 | 0 | 0 | 0 |
| $\left. \left\vert-5 \right. \right\rangle$ | 0 | 0 | 48.8 | 48.7 | 0 | 0 |
| $\left. \left\vert-4 \right. \right\rangle$ | 0 | 0 | 0 | 0 | 40.5 | 40.5 |
| $\left. \left\vert-3 \right. \right\rangle$ | 0.1 | 0.1 | 0 | 0 | 0 | 0 |
| $\left. \left\vert-2 \right. \right\rangle$ | 0 | 0 | 1.2 | 1.2 | 0 | 0 |
| $\left. \left\vert-1 \right. \right\rangle$ | 0 | 0 | 0 | 0 | 9.4 | 9.4 |
| $\left. \left\vert0 \right. \right\rangle$ | 0 | 0 | 0 | 0 | 0 | 0 |
| $\left. \left\vert+ \right.1 \right\rangle$ | 0 | 0 | 0 | 0 | 9.4 | 9.4 |
| $\left. \left\vert+2 \right. \right\rangle$ | 0 | 0 | 1.2 | 1.2 | 0 | 0 |
| $\left. \left\vert+3 \right. \right\rangle$ | 0.1 | 0.1 | 0 | 0 | 0 | 0 |
| $\left. \left\vert+ \right.4 \right\rangle$ | 0 | 0 | 0 | 0 | 40.5 | 40.5 |
| $\left. \left\vert+5 \right. \right\rangle$ | 0 | 0 | 48.7 | 48.8 | 0 | 0 |
| $\left. \left\vert+6 \right. \right\rangle$ | 49.9 | 49.9 | 0 | 0 | 0 | 0 |
|  | **Wavefunction** | | | | | |
| *m_J_* | **State 7**  **(533.712 cm^-1^)** | **State 8**  **(626.426 cm^-1^)** | **State 9**  **(653.244 cm^-1^)** | **State 10**  **(653.269 cm^-1^)** | **State 11**  **(713.125 cm^-1^)** | **State 12**  **(713.156 cm^-1^)** |
| $\left. \left\vert-6 \right. \right\rangle$ | 0.1 | 0.1 | 0 | 0 | 0 | 0 |
| $\left. \left\vert-5 \right. \right\rangle$ | 0 | 0 | 0.9 | 0.9 | 0.3 | 0.3 |
| $\left. \left\vert-4 \right. \right\rangle$ | 0 | 0 | 3.9 | 3.9 | 5.6 | 5.5 |
| $\left. \left\vert-3 \right. \right\rangle$ | 24.2 | 49.9 | 0 | 0 | 0 | 0 |
| $\left. \left\vert-2 \right. \right\rangle$ | 0 | 0 | 31.5 | 31.5 | 17.2 | 17.2 |
| $\left. \left\vert-1 \right. \right\rangle$ | 0 | 0 | 13.7 | 13.7 | 26.9 | 26.9 |
| $\left. \left\vert0 \right. \right\rangle$ | 51.5 | 0 | 0 | 0 | 0 | 0 |
| $\left. \left\vert+ \right.1 \right\rangle$ | 0 | 0 | 13.7 | 13.7 | 26.9 | 26.9 |
| $\left. \left\vert+2 \right. \right\rangle$ | 0 | 0 | 31.5 | 31.5 | 17.2 | 17.2 |
| $\left. \left\vert+3 \right. \right\rangle$ | 24.2 | 49.9 | 0 | 0 | 0 | 0 |
| $\left. \left\vert+ \right.4 \right\rangle$ | 0 | 0 | 3.9 | 3.9 | 5.6 | 5.5 |
| $\left. \left\vert+5 \right. \right\rangle$ | 0 | 0 | 0.9 | 0.9 | 0.3 | 0.3 |
| $\left. \left\vert+6 \right. \right\rangle$ | 0.1 | 0.1 | 0 | 0 | 0 | 0 |
|  | **Wavefunction** |  |  |  |  |  |
| *m_J_* | **State 13**  **(750.234 cm^-1^)** |  |  |  |  |  |
| $\left. \left\vert-6 \right. \right\rangle$ | 0 |  |  |  |  |  |
| $\left. \left\vert-5 \right. \right\rangle$ | 0 |  |  |  |  |  |
| $\left. \left\vert-4 \right. \right\rangle$ | 0 |  |  |  |  |  |
| $\left. \left\vert-3 \right. \right\rangle$ | 25.7 |  |  |  |  |  |
| $\left. \left\vert-2 \right. \right\rangle$ | 0 |  |  |  |  |  |
| $\left. \left\vert-1 \right. \right\rangle$ | 0 |  |  |  |  |  |
| $\left. \left\vert0 \right. \right\rangle$ | 48.5 |  |  |  |  |  |
| $\left. \left\vert+ \right.1 \right\rangle$ | 0 |  |  |  |  |  |
| $\left. \left\vert+2 \right. \right\rangle$ | 0 |  |  |  |  |  |
| $\left. \left\vert+3 \right. \right\rangle$ | 25.7 |  |  |  |  |  |
| $\left. \left\vert+ \right.4 \right\rangle$ | 0 |  |  |  |  |  |
| $\left. \left\vert+5 \right. \right\rangle$ | 0 |  |  |  |  |  |
| $\left. \left\vert+6 \right. \right\rangle$ | 0.1 |  |  |  |  |  |

**Table S12.** Computed energy levels (the ground state is set at zero), composition of the g-tensor (g_x_, g_y_, g_z_) and the main components (>5%) of the wavefunction for each *m_J_* state of the ground-state multiplet ^6^H_15/2_ for the Dy^3+^ in **Ni_2_Dy**, at the CASSCF level.

| **Energy (cm^-1^)** | ***g_x_*** | ***g_y_*** | ***g_z_*** | **Wavefunction** |
| --- | --- | --- | --- | --- |
| 0 | 0.0000 | 0.0000 | 19.1167 | 90.9% \|±15/2>+8.6% \|±9/2> |
| 51.231 | 1.0658 | 1.0663 | 14.3954 | 95.1% \|±11/2> |
| 53.675 | 1.0001 | 1.0004 | 17.0149 | 99.0% \|±13/2> |
| 109.358 | 0.0003 | 0.0005 | 12.0579 | 9.1% \|±15/2>+84.4% \|±9/2> |
| 202.760 | 4.3262 | 4.3271 | 7.8157 | 86.0% \|±5/2>+10.3% \|±1/2> |
| 381.181 | 4.2483 | 4.2489 | 5.3695 | 6.5% \|±7/2>+85.1% \|±5/2> |
| 529.744 | 0.0019 | 0.0044 | 4.3464 | 93% \|±3/2> |
| 623.798 | 10.4067 | 10.4009 | 1.1780 | 85.1% \|±1/2>+7% \|±7/2> |

**Table S13.** Computed energy levels (the ground state is set at zero), wavefunction *m_J_* composition, of the ground-state multiplet ^5^I_8_ for the Ho^3+^ in **Ni_2_Ho**, at the CASSCF level.

|  | **Wavefunction** | | | | | |
| --- | --- | --- | --- | --- | --- | --- |
| *m_J_* | **State 1**  **(0 cm^-1^)** | **State 2**  **(0 cm^-1^)** | **State 3**  **(74.727 cm^-1^)** | **State 4**  **(74.727 cm^-1^)** | **State 5**  **(93.483 cm^-1^)** | **State 6**  **(95.761 cm^-1^)** |
| $\left. \left\vert-6 \right. \right\rangle$ | 0 | 0 | 23.1 | 23.1 | 0 | 0 |
| $\left. \left\vert-5 \right. \right\rangle$ | 40.8 | 40.8 | 0 | 0 | 0 | 0 |
| $\left. \left\vert-4 \right. \right\rangle$ | 0 | 0 | 0 | 0 | 45.8 | 46.4 |
| $\left. \left\vert-3 \right. \right\rangle$ | 0 | 0 | 26.8 | 26.8 | 0 | 0 |
| $\left. \left\vert-2 \right. \right\rangle$ | 9.1 | 9.1 | 0 | 0 | 0 | 0 |
| $\left. \left\vert-1 \right. \right\rangle$ | 0 | 0 | 0 | 0 | 4.2 | 3.5 |
| $\left. \left\vert0 \right. \right\rangle$ | 0 | 0 | 0 | 0 | 0 | 0 |
| $\left. \left\vert+ \right.1 \right\rangle$ | 0.1 | 0.1 | 0.1 | 0.1 | 0 | 0 |
| $\left. \left\vert+2 \right. \right\rangle$ | 0 | 0 | 0 | 0 | 0 | 0.1 |
| $\left. \left\vert+3 \right. \right\rangle$ | 0.1 | 0.1 | 0.1 | 0.1 | 0 | 0 |
| $\left. \left\vert+ \right.4 \right\rangle$ | 0 | 0 | 0 | 0 | 0 | 0 |
| $\left. \left\vert+5 \right. \right\rangle$ | 0 | 0 | 0 | 0 | 4.2 | 3.5 |
| $\left. \left\vert+6 \right. \right\rangle$ | 9.1 | 9.1 | 0 | 0 | 0 | 0 |
|  | **Wavefunction** | | | | | |
| *m_J_* | **State 7**  **(268.629 cm^-1^)** | **State 8**  **(293.324 cm^-1^)** | **State 9**  **(293.326 cm^-1^)** | **State 10**  **(336.386 cm^-1^)** | **State 11**  **(336.399 cm^-1^)** | **State 12**  **(342.299 cm^-1^)** |
| $\left. \left\vert-6 \right. \right\rangle$ | 0 | 0.4 | 0.4 | 0.6 | 0.6 | 0 |
| $\left. \left\vert-5 \right. \right\rangle$ | 0 | 3.6 | 3.6 | 1.8 | 1.8 | 0 |
| $\left. \left\vert-4 \right. \right\rangle$ | 1.7 | 0 | 0 | 0 | 0 | 4.2 |
| $\left. \left\vert-3 \right. \right\rangle$ | 0 | 0.1 | 0.1 | 0.3 | 0.3 | 0 |
| $\left. \left\vert-2 \right. \right\rangle$ | 0 | 11.8 | 11.8 | 7 | 7 | 0 |
| $\left. \left\vert-1 \right. \right\rangle$ | 16.8 | 0 | 0 | 0 | 0 | 45.8 |
| $\left. \left\vert0 \right. \right\rangle$ | 0 | 2.3 | 2.4 | 40.2 | 40.2 | 0 |
| $\left. \left\vert+ \right.1 \right\rangle$ | 0 | 31.7 | 31.7 | 0 | 0.1 | 0 |
| $\left. \left\vert+2 \right. \right\rangle$ | 63.1 | 0 | 0 | 0 | 0 | 0 |
| $\left. \left\vert+3 \right. \right\rangle$ | 0 | 31.7 | 31.7 | 0 | 0.1 | 0 |
| $\left. \left\vert+ \right.4 \right\rangle$ | 0 | 2.3 | 2.4 | 40.2 | 40.2 | 0 |
| $\left. \left\vert+5 \right. \right\rangle$ | 16.8 | 0 | 0 | 0 | 0 | 45.8 |
| $\left. \left\vert+6 \right. \right\rangle$ | 0 | 11.8 | 11.8 | 7 | 7 | 0 |

**Table S13. (continuation)** Computed energy levels (the ground state is set at zero), wavefunction *m_J_* composition, of the ground-state multiplet ^5^I_8_ for the Ho^3+^ in **Ni_2_Ho**, at the CASSCF level.

|  | **Wavefunction** | | | | |
| --- | --- | --- | --- | --- | --- |
| *m_J_* | **State 13**  **(386.757 cm^-1^)** | **State 14**  **(386.758 cm^-1^)** | **State 15**  **(413.294 cm^-1^)** | **State 16**  **(413.303 cm^-1^)** | **State 17**  **(434.889 cm^-1^)** |
| $\left. \left\vert-6 \right. \right\rangle$ | 25.6 | 25.6 | 0.2 | 0.2 | 0 |
| $\left. \left\vert-5 \right. \right\rangle$ | 0 | 0 | 3.7 | 3.7 | 0 |
| $\left. \left\vert-4 \right. \right\rangle$ | 0 | 0 | 0 | 0 | 1.8 |
| $\left. \left\vert-3 \right. \right\rangle$ | 22.5 | 22.5 | 0.3 | 0.3 | 0 |
| $\left. \left\vert-2 \right. \right\rangle$ | 0.2 | 0.2 | 21.9 | 21.9 | 0 |
| $\left. \left\vert-1 \right. \right\rangle$ | 0 | 0 | 0 | 0 | 29.7 |
| $\left. \left\vert0 \right. \right\rangle$ | 1.6 | 1.6 | 5.8 | 5.8 | 0 |
| $\left. \left\vert+ \right.1 \right\rangle$ | 0 | 0 | 18 | 18 | 0 |
| $\left. \left\vert+2 \right. \right\rangle$ | 0 | 0 | 0 | 0 | 36.8 |
| $\left. \left\vert+3 \right. \right\rangle$ | 0 | 0 | 18 | 18 | 0 |
| $\left. \left\vert+ \right.4 \right\rangle$ | 1.6 | 1.6 | 5.8 | 5.8 | 0 |
| $\left. \left\vert+5 \right. \right\rangle$ | 0 | 0 | 0 | 0 | 29.7 |
| $\left. \left\vert+6 \right. \right\rangle$ | 0.2 | 0.2 | 0 | 0 | 0 |

**Table S14.** Computed energy levels (the ground state is set at zero), composition of the g-tensor (g_x_, g_y_, g_z_) and the main components (>10%) of the wavefunction for each *m_J_* state of the ground-state multiplet ^4^I_15/2_ for the Er^3+^ in **Ni_2_Er**, at the CASSCF level.

| **Energy (cm^-1^)** | ***g_x_*** | ***g_y_*** | ***g_z_*** | **Wavefunction** |
| --- | --- | --- | --- | --- |
| 0 | 0.0005 | 0.0007 | 14.8290 | 59.6% \|±15/2>+37.7% \|±9/2> |
| 23.861 | 8.2010 | 8.2007 | 3.9679 | 42.4% \|±7/2>+18% \|±5/2>+30.8% \|±1/2> |
| 107.965 | 7.6562 | 7.6484 | 0.4107 | 27.6% \|±7/2>+58.6% \|±5/2> |
| 126.621 | 0.0040 | 0.0049 | 3.8434 | 96.6% \|±3/2> |
| 186.510 | 8.9992 | 8.9977 | 1.1494 | 10% \|±7/2>+66.8% \|±1/2>+17.9% \|±5/2> |
| 348.656 | 0.2261 | 0.2262 | 12.7666 | 94.5% \|±11/2> |
| 368.493 | 0.0001 | 0.0002 | 13.3762 | 27.1% \|±15/2>+62.2% \|±9/2> |
| 448.854 | 0.2136 | 0.2137 | 14.0091 | 79.4% \|±13/2>+20.2% \|±7/2> |

**Table S15.** Computed energy levels (the ground state is set at zero), composition of the g-tensor (g_x_, g_y_, g_z_) and the main components (>10%) of the wavefunction for each *m_J_* state of the ground-state multiplet ^2^F_7/2_ for the Yb^3+^ in **Ni_2_Yb**, at the CASSCF level.

| **Energy (cm^-1^)** | ***g_x_*** | ***g_y_*** | ***g_z_*** | **Wavefunction** |
| --- | --- | --- | --- | --- |
| 0 | 1.4836 | 1.4893 | 4.1715 | 78.5% \|±5/2>+20.3% \|±1/2> |
| 94.907 | 0.0021 | 0.0046 | 3.4112 | 100% \|±3/2> |
| 260.596 | 0.6572 | 0.6584 | 4.9625 | 67.1% \|±7/2>+21.8% \|±1/2>+11.2% \|±5/2> |
| 348.383 | 3.7298 | 3.7285 | 2.6107 | 31.8% \|±7/2>+10.2% \|±5/2>+>+58% \|±1/2> |

**Table S16.** Computed energy levels (the ground state is set at zero), wavefunction *m_J_* composition, of the ground-state multiplet ^7^F_6_ for the Tb^3+^ in **Zn_2_Tb**, at the CASSCF level.

|  | **Wavefunction** | | | | | |
| --- | --- | --- | --- | --- | --- | --- |
| *m_J_* | **State 1**  **(0 cm^-1^)** | **State 2**  **(0.013cm^-1^)** | **State 3**  **(267.692 cm^-1^)** | **State 4**  **(267.692 cm^-1^)** | **State 5**  **(451.005 cm^-1^)** | **State 6**  **(451.005 cm^-1^)** |
| $\left. \left\vert-6 \right. \right\rangle$ | 49.9 | 49.9 | 0 | 0 | 0 | 0 |
| $\left. \left\vert-5 \right. \right\rangle$ | 0 | 0 | 48.8 | 48.8 | 0 | 0 |
| $\left. \left\vert-4 \right. \right\rangle$ | 0 | 0 | 0 | 0 | 40.6 | 40.4 |
| $\left. \left\vert-3 \right. \right\rangle$ | 0.1 | 0.1 | 0 | 0 | 0 | 0 |
| $\left. \left\vert-2 \right. \right\rangle$ | 0 | 0 | 1.2 | 1.2 | 0.1 | 0.1 |
| $\left. \left\vert-1 \right. \right\rangle$ | 0 | 0 | 0 | 0 | 9.5 | 9.4 |
| $\left. \left\vert0 \right. \right\rangle$ | 0 | 0 | 0 | 0 | 0 | 0 |
| $\left. \left\vert+ \right.1 \right\rangle$ | 0 | 0 | 0 | 0 | 9.4 | 9.5 |
| $\left. \left\vert+2 \right. \right\rangle$ | 0 | 0 | 1.2 | 1.2 | 0.1 | 0 |
| $\left. \left\vert+3 \right. \right\rangle$ | 0.1 | 0.1 | 0 | 0 | 0 | 0 |
| $\left. \left\vert+ \right.4 \right\rangle$ | 0 | 0 | 0 | 0 | 40.4 | 40.6 |
| $\left. \left\vert+5 \right. \right\rangle$ | 0 | 0 | 48.8 | 48.8 | 0 | 0 |
| $\left. \left\vert+6 \right. \right\rangle$ | 49.9 | 49.9 | 0 | 0 | 0 | 0 |
|  | **Wavefunction** | | | | | |
| *m_J_* | **State 7**  **(513.788 cm^-1^)** | **State 8**  **(596.577 cm^-1^)** | **State 9**  **(617.841 cm^-1^)** | **State 10**  **(617.866 cm^-1^)** | **State 11**  **(667.866 cm^-1^)** | **State 12**  **(667.884 cm^-1^)** |
| $\left. \left\vert-6 \right. \right\rangle$ | 0.1 | 0.1 | 0 | 0 | 0 | 0 |
| $\left. \left\vert-5 \right. \right\rangle$ | 0 | 0 | 0.9 | 0.9 | 0.3 | 0.3 |
| $\left. \left\vert-4 \right. \right\rangle$ | 0 | 0 | 4.1 | 4.1 | 5.4 | 5.4 |
| $\left. \left\vert-3 \right. \right\rangle$ | 23.9 | 49.9 | 0 | 0 | 0 | 0 |
| $\left. \left\vert-2 \right. \right\rangle$ | 0 | 0 | 31 | 31 | 17.7 | 17.7 |
| $\left. \left\vert-1 \right. \right\rangle$ | 0 | 0 | 14 | 14 | 26.5 | 26.5 |
| $\left. \left\vert0 \right. \right\rangle$ | 52.2 | 0 | 0 | 0 | 0 | 0 |
| $\left. \left\vert+ \right.1 \right\rangle$ | 0 | 0 | 14 | 14 | 26.5 | 26.5 |
| $\left. \left\vert+2 \right. \right\rangle$ | 0 | 0 | 31 | 31 | 17.7 | 17.7 |
| $\left. \left\vert+3 \right. \right\rangle$ | 23.9 | 49.9 | 0 | 0 | 0 | 0 |
| $\left. \left\vert+ \right.4 \right\rangle$ | 0 | 0 | 4.1 | 4.1 | 5.4 | 5.4 |
| $\left. \left\vert+5 \right. \right\rangle$ | 0 | 0 | 0.9 | 0.9 | 0.3 | 0.3 |
| $\left. \left\vert+6 \right. \right\rangle$ | 0.1 | 0.1 | 0 | 0 | 0 | 0 |
|  | **Wavefunction** |  |  |  |  |  |
| *m_J_* | **State 13**  **(699.213 cm^-1^)** |  |  |  |  |  |
| $\left. \left\vert-6 \right. \right\rangle$ | 0 |  |  |  |  |  |
| $\left. \left\vert-5 \right. \right\rangle$ | 0 |  |  |  |  |  |
| $\left. \left\vert-4 \right. \right\rangle$ | 0 |  |  |  |  |  |
| $\left. \left\vert-3 \right. \right\rangle$ | 26.1 |  |  |  |  |  |
| $\left. \left\vert-2 \right. \right\rangle$ | 0 |  |  |  |  |  |
| $\left. \left\vert-1 \right. \right\rangle$ | 0 |  |  |  |  |  |
| $\left. \left\vert0 \right. \right\rangle$ | 47.8 |  |  |  |  |  |
| $\left. \left\vert+ \right.1 \right\rangle$ | 0 |  |  |  |  |  |
| $\left. \left\vert+2 \right. \right\rangle$ | 0 |  |  |  |  |  |
| $\left. \left\vert+3 \right. \right\rangle$ | 26.1 |  |  |  |  |  |
| $\left. \left\vert+ \right.4 \right\rangle$ | 0 |  |  |  |  |  |
| $\left. \left\vert+5 \right. \right\rangle$ | 0 |  |  |  |  |  |
| $\left. \left\vert+6 \right. \right\rangle$ | 0 |  |  |  |  |  |

**Table S17.** Computed energy levels (the ground state is set at zero), composition of the g-tensor (g_x_, g_y_, g_z_) and the main components (>5%) of the wavefunction for each *m_J_* state of the ground-state multiplet ^6^H_15/2_ for the Dy^3+^ in **Zn_2_Dy**, at the CASSCF level.

| **Energy (cm^-1^)** | ***g_x_*** | ***g_y_*** | ***g_z_*** | **Wavefunction** |
| --- | --- | --- | --- | --- |
| 0 | 0.0000 | 0.0000 | 19.3111 | 93.2% \|±15/2>+6.6% \|±9/2> |
| 39.759 | 0.7480 | 0.7488 | 17.085 | 99.1% \|±13/2> |
| 42.416 | 0.7070 | 0.7072 | 14.1738 | 96.0% \|±11/2> |
| 102.956 | 0.0001 | 0.0005 | 11.9474 | 6.8% \|±15/2>+87.5% \|±9/2>+5.7% \|±3/2> |
| 201.832 | 3.8746 | 3.8751 | 8.0562 | 87.3% \|±7/2>+8.9% \|±1/2> |
| 369.017 | 3.7897 | 3.7923 | 5.5552 | 5.1% \|±7/2>+87.1% \|±5/2> |
| 510.650 | 0.0000 | 0.0048 | 4.2737 | 923% \|±3/2> |
| 599.238 | 10.4295 | 10.4247 | 1.1885 | 83.2% \|±1/2>+6.9% \|±5/2> |

**Table S18**: Crystal field Hamiltonian is given as ${\hat{\boldsymbol{H}}}_{\boldsymbol{CF}}\boldsymbol{=}\sum_{\boldsymbol{k,q}} \boldsymbol{B}_{\boldsymbol{k}}^{\boldsymbol{q}}\boldsymbol{O}_{\boldsymbol{k}}^{\boldsymbol{q}}$ and the extended Stevens operator coefficients $\boldsymbol{B}_{\boldsymbol{k}}^{\boldsymbol{q}}$ are extracted from CASSCF calculations for the **Ni_2_Ln** series.

|  |  | $B_{k}^{q}$ | | | | |
| --- | --- | --- | --- | --- | --- | --- |
| *k* | *q* | **Tb** | **Dy** | **Ho** | **Er** | **Yb** |
| 2 | -2 | 9.24362E-5 | -2.63466E-4 | -1.13525E-4 | -5.88426E-5 | 1.49126E-4 |
| 2 | -1 | **0.00101** | **-0.00209** | -2.71263E-4 | **0.00165** | **0.02807** |
| **2** | **0** | **-6.19033** | **-3.19596** | **-1.13509** | **0.84386** | **1.93003** |
| 2 | 1 | 5.94304E-4 | 2.76075E-4 | **0.00208** | **-0.00109** | **-0.00897** |
| 2 | 2 | -9.58283E-5 | -7.47917E-4 | 1.72489E-4 | 2.20863E-4 | **-0.00492** |
| 4 | -4 | -1.52529E-5 | 2.06872E-6 | 1.20946E-5 | 6.207E-6 | **0.00188** |
| 4 | -3 | **0.27635** | **-0.1031** | **-0.05708** | **0.03307** | **-2.40239** |
| 4 | -2 | -9.54339E-6 | 1.06139E-5 | -6.20433E-6 | 1.17892E-5 | -9.1664E-5 |
| 4 | -1 | -1.74403E-6 | 1.6368E-5 | 5.40413E-6 | -2.62456E-5 | **0.00498** |
| 4 | 0 | **-0.01265** | **0.00805** | **0.00359** | **-0.00612** | **0.1731** |
| 4 | 1 | -1.74899E-5 | -3.43016E-6 | -2.38328E-5 | 1.41213E-5 | **-0.00224** |
| 4 | 2 | 3.21572E-6 | 1.35828E-5 | 3.87278E-6 | 2.89452E-6 | **0.00208** |
| 4 | 3 | **-0.01747** | **0.04934** | **0.01248** | **0.08132** | **0.99318** |
| 4 | 4 | -2.88703E-5 | -2.23175E-5 | -4.75991E-7 | -9.2626E-6 | **0.00158** |
| 6 | -6 | 2.64988E-5 | -8.65789E-5 | 4.06006E-5 | 9.63058E-5 | **-0.00647** |
| 6 | -5 | 6.26299E-7 | -2.2764E-7 | 1.91521E-7 | 5.50806E-7 | -3.57816E-5 |
| 6 | -4 | -5.62978E-7 | -1.03828E-7 | 4.72129E-7 | 3.48284E-7 | -1.64861E-4 |
| 6 | -3 | **-0.00148** | 8.51089E-4 | **-0.00103** | 6.34943E-4 | **0.09133** |
| 6 | -2 | -3.46822E-7 | 5.47704E-8 | -2.42957E-7 | 4.6175E-7 | 1.10507E-5 |
| 6 | -1 | 1.48723E-7 | -1.15595E-7 | 1.11453E-7 | -4.18541E-7 | -2.2118E-4 |
| 6 | 0 | 8.69418E-5 | -3.27553E-5 | 3.37437E-5 | -5.45561E-5 | **-0.00287** |
| 6 | 1 | 7.99594E-8 | 3.33942E-8 | -3.16988E-7 | 1.05467E-7 | 1.14847E-4 |
| 6 | 2 | 8.38921E-7 | -6.17319E-8 | 1.67518E-7 | -8.17307E-8 | -2.41179E-4 |
| 6 | 3 | 9.35851E-5 | -4.07083E-4 | 2.25073E-4 | **0.00156** | **-0.03772** |
| 6 | 4 | -6.98861E-7 | -4.40477E-7 | 2.53756E-8 | -2.98591E-7 | -1.58598E-4 |
| 6 | 5 | 3.1539E-7 | 6.01531E-7 | 2.82987E-7 | 1.39257E-8 | 2.49802E-5 |
| 6 | 6 | 2.08879E-4 | -6.97997E-5 | 8.82076E-5 | 9.87054E-5 | **-0.00649** |

**Table S19**: Crystal field Hamiltonian is given as ${\hat{\boldsymbol{H}}}_{\boldsymbol{CF}}\boldsymbol{=}\sum_{\boldsymbol{k,q}} \boldsymbol{B}_{\boldsymbol{k}}^{\boldsymbol{q}}\boldsymbol{O}_{\boldsymbol{k}}^{\boldsymbol{q}}$ and the extended Stevens operator coefficients $\boldsymbol{B}_{\boldsymbol{k}}^{\boldsymbol{q}}$ are extracted from CASSCF calculations for **Zn_2_Ln** systems.

|  |  | $B_{k}^{q}$ | |
| --- | --- | --- | --- |
| *k* | *q* | **Tb** | **Dy** |
| 2 | -2 | -2.57329E-5 | -9.79834E-4 |
| 2 | -1 | 5.12799E-4 | **-0.01018** |
| **2** | **0** | **-5.80346** | **-3.15528** |
| 2 | 1 | 6.00224E-4 | **0.00148** |
| 2 | 2 | 8.39065E-7 | -5.10639E-4 |
| 4 | -4 | 4.38255E-7 | 1.12384E-5 |
| 4 | -3 | **0.20906** | **-0.0632** |
| 4 | -2 | -3.49222E-6 | 3.11336E-5 |
| 4 | -1 | -7.39134E-6 | 1.05007E-4 |
| 4 | 0 | **-0.01281** | **0.00809** |
| 4 | 1 | -8.65026E-6 | -1.99122E-5 |
| 4 | 2 | -5.81431E-7 | 2.93773E-5 |
| 4 | 3 | **-0.12777** | **0.08277** |
| 4 | 4 | -1.27691E-6 | -1.10302E-5 |
| 6 | -6 | 1.40432E-4 | -9.00034E-5 |
| 6 | -5 | -3.18611E-9 | 2.9843E-7 |
| 6 | -4 | -7.0857E-9 | -9.64484E-7 |
| 6 | -3 | **-0.00103** | 5.00029E-4 |
| 6 | -2 | 3.51775E-8 | -3.75004E-7 |
| 6 | -1 | 5.13298E-8 | -7.57974E-7 |
| 6 | 0 | 7.68168E-5 | -2.98517E-5 |
| 6 | 1 | 5.9951E-8 | 1.58229E-7 |
| 6 | 2 | 6.07663E-9 | -5.36218E-7 |
| 6 | 3 | 6.32492E-4 | -6.54465E-4 |
| 6 | 4 | 2.38779E-8 | -6.5904E-7 |
| 6 | 5 | -1.34414E-8 | 9.04603E-7 |
| 6 | 6 | 7.19801E-5 | 2.45175E-5 |

**Table S20.** Exchange interaction operating within the **Ni_2_Ln** systems as obtained from the simultaneous fits of the χ_M_*T*(*T*) and *M*(*H*) data.

| Fitting parameters for ***J*_Ni-Ni_** and ***J*_Ni-Ln_** | | | | | | |
| --- | --- | --- | --- | --- | --- | --- |
| **Parameter** | **Y** | **Tb** | **Dy** | **Ho** | **Er** | **Yb** |
| ***g*_Ni_** | 2.292(1) | 2.491(2) | 2.073(3) | 2.118(2) | 2.415(3) | 2.269(2) |
| ***J*_Ni-Ln_** (cm^-1^) | **-** | **0.14(1)** | **0.31(3)** | **0.17(1)** | **-0.0061(5)** | **-0.08(6)** |
| ***J*_Ni-Ni_** (cm^-1^) | -0.106(1) | -0.42(9) | -0.06(4) | 0.15(2) | 0.43(2) | 0.28(4) |
| ***D*** (cm^-1^)*^a^* | -1.6(2) | -1.2225 | -2.0925 | -2.508 | -3.003 | -3.2085 |
| ***E*** (cm^-1^) *^b^* | - | -0.0005 | -0.0005 | -0.002 | -0.002 | -0.0005 |
| ***R^2^*** | 0.24% | 2.31% | 1.27% | 7.63% | 5.32% | 0.14% |

*^a^* This parameter was fitted just for the **Ni_2_Y**, while for the remaining **Ni_2_Ln** systems, this parameter was kept fixed to the one obtained from CASSCF calculations.

*^b^* This parameter was kept fixed to zero **Ni_2_Y**, while for the remaining **Ni_2_Ln** systems, it was fixed to the one obtained from CASSCF calculations.

**Table S21.** Molecular systems with reported τ_QTM_

| **No.** | **Molecular Formula** | **τ_QTM_ (s)** | **References** |
| --- | --- | --- | --- |
| 1. | [Dy(Cp^ttt^)_2_][B(C_6_F_5_)_4_] | 2.5 × 10^4^ | *Science* 362, 2018, 1400–1403 |
| 2. | Tb_2_@C_80_(CH_2_Ph) | 6.50 × 10^4^ | *Nat*. *Commun*. 2019, **10**, 571 |
| 3. | Tb_2_@C_80_(CF_3_) | 3.56 × 10^4^ | *J. Am. Chem. Soc*. 2021, **143**, 18139-18149 |
| 4. | Tb_2_@C_79_N | 1.66 × 10^4^ | *Angew. Chem., Int. Ed*. 2019, **58**, 5891-5896 |
| 5. | [K(crypt-222][(Cp^Me4H^_2_Tb)_2_(µ-N·_2_)] | 4.00 × 10^4^ | *Nat. Commun*. 2017, **8**, 2144 |
| 6. | Dy_2_ | 2.43 × 10^4^ | *J. Am. Chem. Soc*. 2024, **146**, 18899−18904 |
| 7. | {Cr_3_Dy_3_} | 6 × 10^7^ | *Matter* 2022, **5**, 3485–3498 |


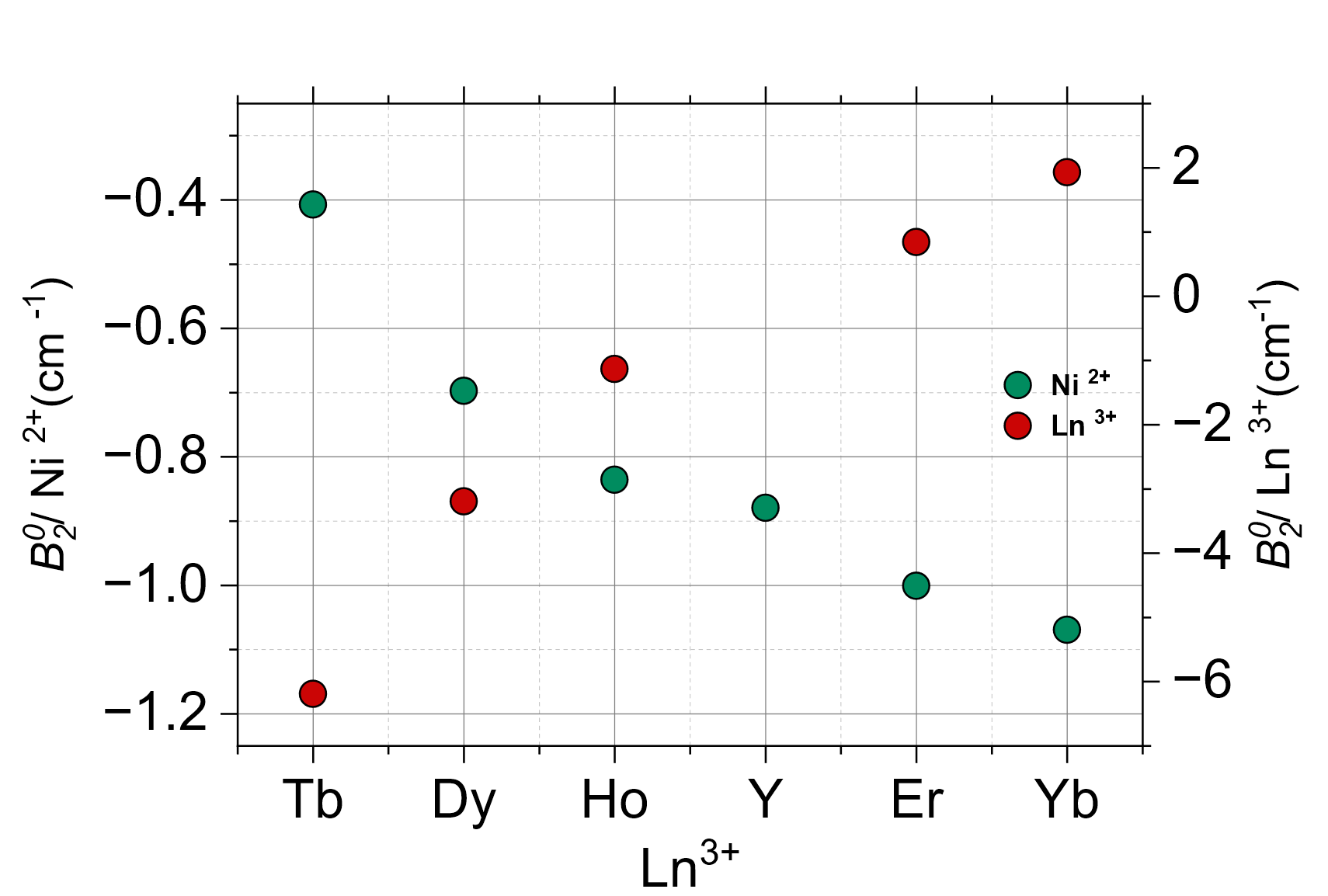


Figure S4. CASSCF calculated $\boldsymbol{B}_{\boldsymbol{2}}^{\boldsymbol{0}}$parameters for the Ni^2+^ and Ln^3+^ in the Ni_2_Ln series.


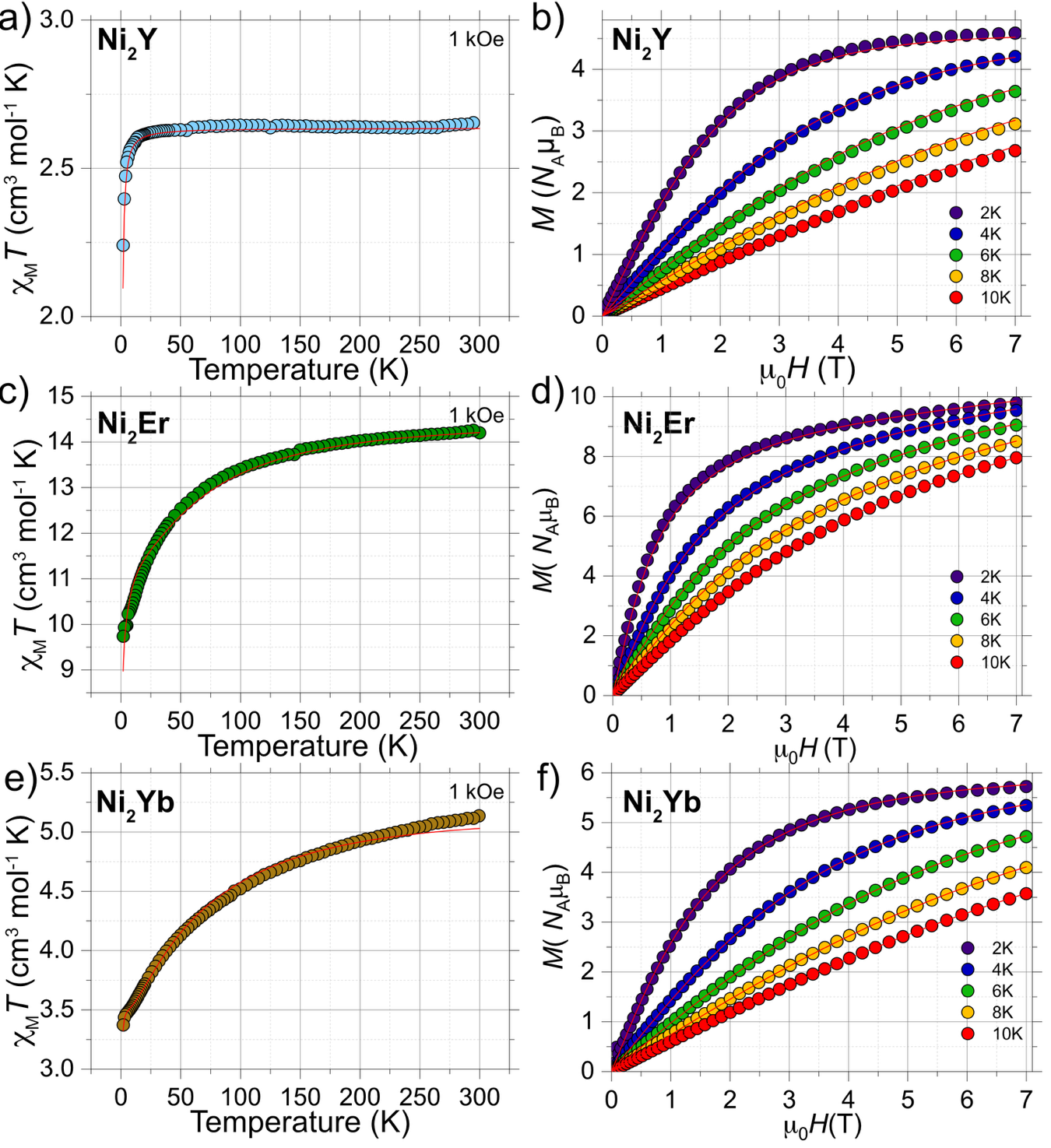


Figure S5. χ_M_*T*(*T*) and *M*(*H*) for Ni_2_Y (a, b), Ni_2_Er (c, d) and Ni_2_Yb (e,f). The solid lines are fits employing the CF parameters obtained from CASSCF calculations and an interaction between the Ni⋅⋅⋅Ln and Ni⋅⋅⋅Ni (see Table S20).


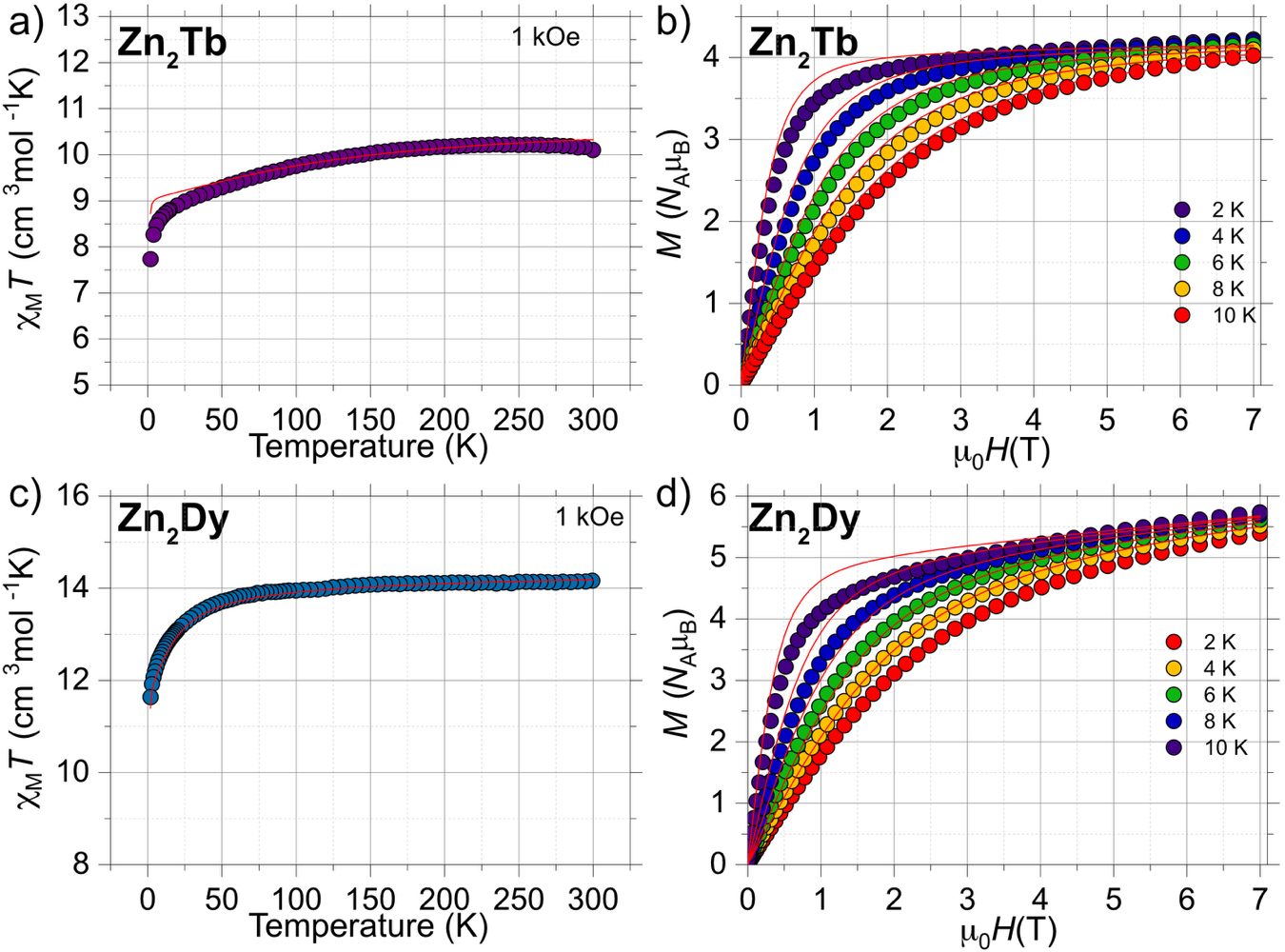


Figure S6. χ_M_*T*(*T*) and *M*(*H*) for Zn_2_Tb (a, b) and Zn_2_Dy (c, d). The solid lines are fits employing the CF parameters obtained from CASSCF calculations. The CASSCF χ_M_*T*(*T*) and *M*(*H*) were scaled up by 1.145 for the Zn_2_Tb data.


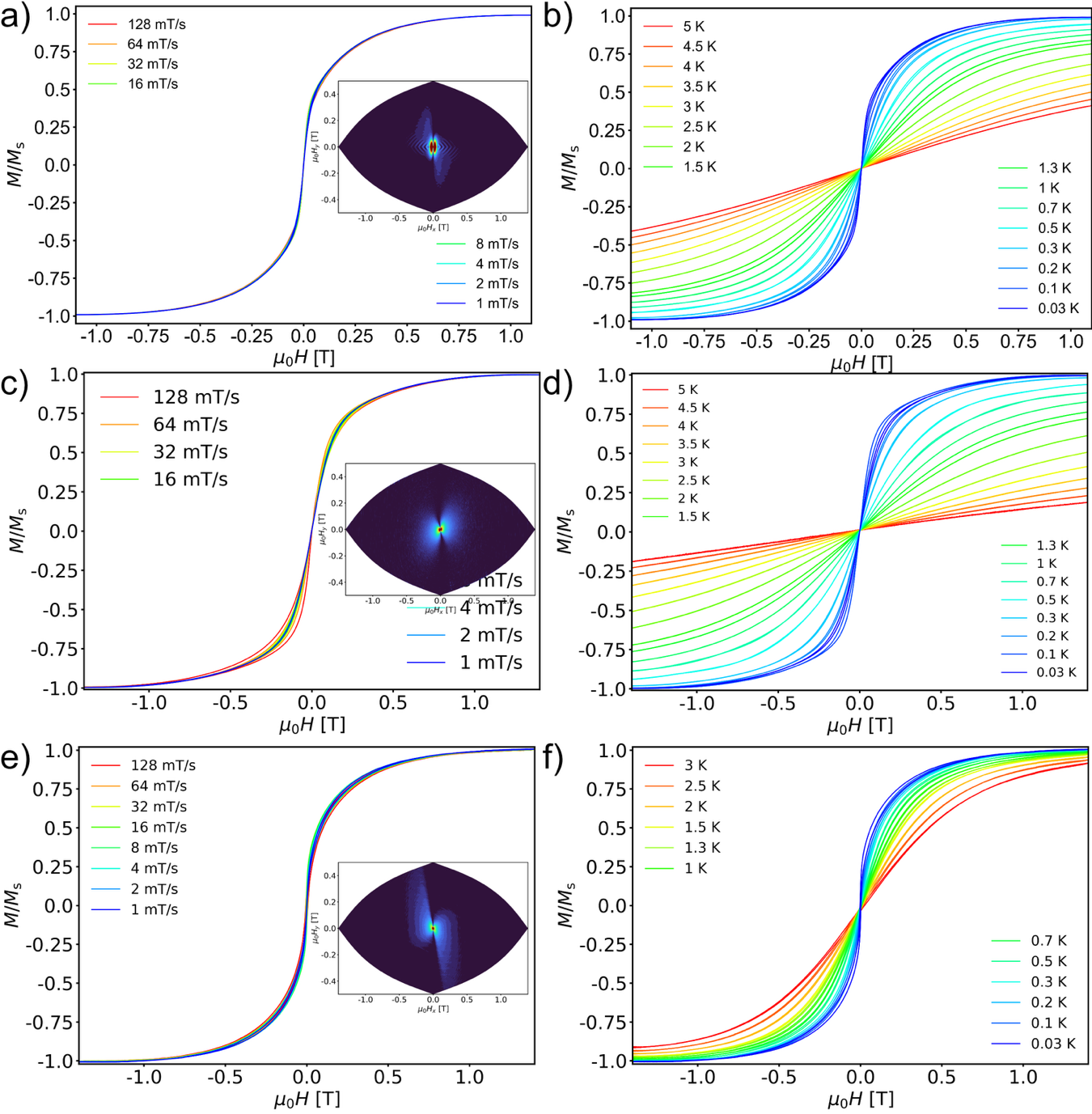


Figure S7. µSQUID loops collected in a single crystal of Ni_2_Er, Ni_2_Yb, and Zn_2_Tb with the applied field along the easy axis of the crystal. Field sweep study at 30 mK and sweep rates from 128 down to 1 mT/s for Ni_2_Er (a), Ni_2_Yb (c) and Zn_2_Tb (e); Angular map highlighting the easy axis for the µSQUID studies (insets); The loops were collected with a sweep rate of 64 mT/s from 30 mK; Temperature-dependent loops with a sweep rate of 16 mT/s from 30 mK up to 5 K for Ni_2_Er (b), Ni_2_Yb (d) and Zn_2_Tb (f)


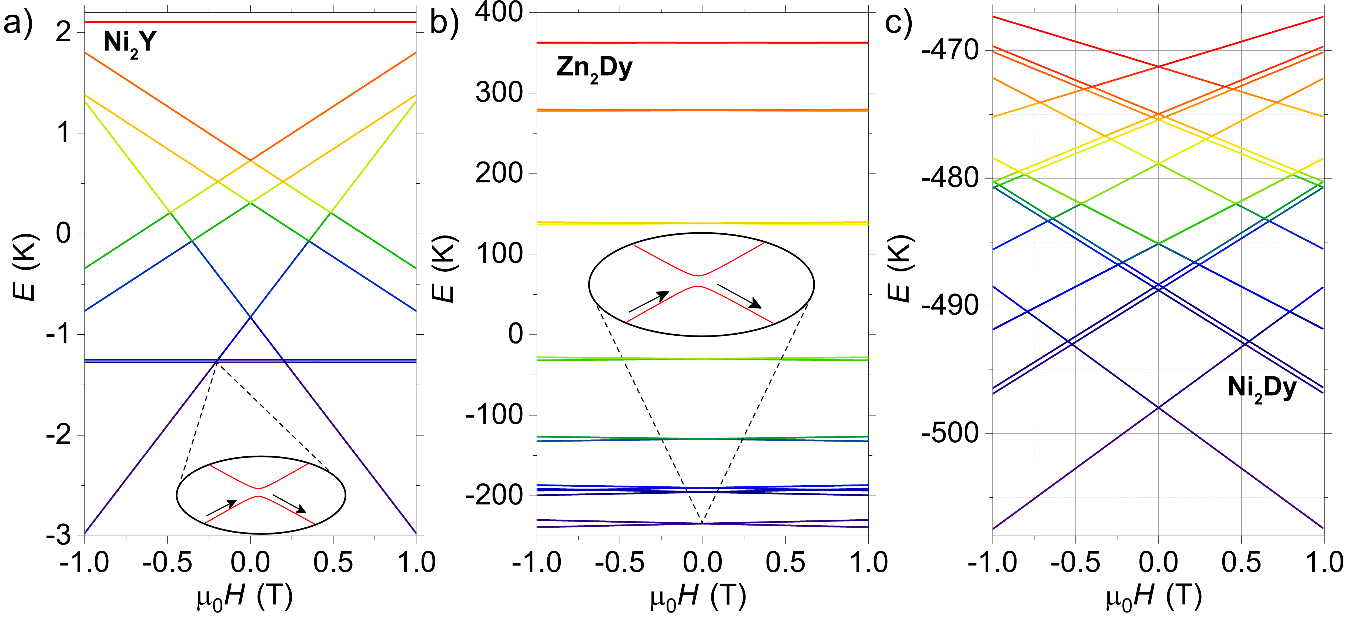


Figure S8. Calculated Zeeman diagrams for the Ni_2_Y (a), Zn_2_Dy and Ni_2_Dy systems, highlighting the different ground and excited states characteristics. The insets in (a) and (b) highlight the strong QTM operating in both systems, based on µSQUID results.

**References**

1. Sheldrick, G. SHELXT: Integrating space group determination and structure solution. Acta Crystallographica Section A Foundations and Advances 2014, 70, C1437-C1437.

2. Dolomanov, O. V.; Bourhis, L. J.; Gildea, R. J.; Howard, J. A. K.; Puschmann, H. OLEX2: a complete structure solution, refinement and analysis program. Journal of Applied Crystallography 2009, 42 (2), 339-341.
